# IMGT^®^ Biocuration and Analysis of the Rhesus Monkey IG Loci

**DOI:** 10.3390/vaccines10030394

**Published:** 2022-03-03

**Authors:** Viviane Nguefack Ngoune, Morgane Bertignac, Maria Georga, Ariadni Papadaki, Alexandre Albani, Géraldine Folch, Joumana Jabado-Michaloud, Véronique Giudicelli, Patrice Duroux, Marie-Paule Lefranc, Sofia Kossida

**Affiliations:** IMGT^®^, The International ImMunoGeneTics Information System^®^, Institute of Human Genetics (IGH), National Center for Scientific Research (CNRS), University of Montpellier (UM), 34000 Montpellier, France; viviane.nguefack-ngoune@igh.cnrs.fr (V.N.N.); morgane_bertignac@hotmail.fr (M.B.); maria.georga@igh.cnrs.fr (M.G.); ariadni.papadaki@igh.cnrs.fr (A.P.); alexandre.albani@igh.cnrs.fr (A.A.); geraldine.folch@igh.cnrs.fr (G.F.); joumana.michaloud@igh.cnrs.fr (J.J.-M.); veronique.giudicelli@igh.cnrs.fr (V.G.); patrice.duroux@igh.cnrs.fr (P.D.); marie-paule.lefranc@outlook.fr (M.-P.L.)

**Keywords:** IMGT, immunoinformatics, immunogenetics, immunoglobulins, IGH locus, IGK locus, IGL locus, *Macaca mulatta*

## Abstract

The adaptive immune system, along with the innate immune system, are the two main biological processes that protect an organism from pathogens. The adaptive immune system is characterized by the specificity and extreme diversity of its antigen receptors. These antigen receptors are the immunoglobulins (IG) or antibodies of the B cells and the T cell receptors (TR) of the T cells. The IG are proteins that have a dual role in immunity: they recognize antigens and trigger elimination mechanisms, to rid the body of foreign cells. The synthesis of the immunoglobulin heavy and light chains requires gene rearrangements at the DNA level in the IGH, IGK, and IGL loci. The rhesus monkey (*Macaca mulatta*) is one of the most widely used nonhuman primate species in biomedical research. In this manuscript, we provide a thorough analysis of the three IG loci of the Mmul_10 assembly of rhesus monkey, integrating IMGT previously existing data. Detailed characterization of IG genes includes their localization and position in the loci, the determination of the allele functionality, and the description of the regulatory elements of their promoters as well as the sequences of the conventional recombination signals (RS). This complete annotation of the genomic IG loci of Mmul_10 assembly and the highly detailed IG gene characterization could be used as a model, in additional rhesus monkey assemblies, for the analysis of the IG allelic polymorphism and structural variation, which have been described in rhesus monkeys.

## 1. Introduction

The immune system, known as the biological defense of the body, is made up of two parts: the innate immune system or non-specific immune system and the adaptive immune system. The adaptive immune system, also referred to as the acquired immune system, is characterized by the remarkable specificity and the extreme diversity of their antigen receptors [1]. These antigen receptors of the adaptive immune response are the immunoglobulins (IG) or antibodies of the B cells [2] and the T cell receptors (TR) of the T cells [3]. IG are proteins that have a dual role in immunity: they recognize antigens on the surface of foreign bodies such as bacteria and viruses and trigger elimination mechanisms such as cell lysis and phagocytosis, to rid the body of these cells and particles [2]. An IG comprises two identical heavy chains (IGH), associated with two identical light chains, kappa (IGK) or lambda (IGL). The synthesis of the immunoglobulin heavy and light chains requires gene rearrangements at the DNA level in the IGH, IGK, and IGL loci during the B cell differentiation [4]. The IGH locus comprises four types of genes, variable (V), diversity (D), joining (J), and constant (C) [5] whereas the IGK [6] and IGL [7] loci lack the D genes. In human, the immunoglobulins comprise a variable domain and a constant region which is composed of one constant domain (IG light chains) or three or four constant domains for IG heavy chain [8]. The variable domain is the result of one rearrangement between variable (V) and joining (J) genes for IGL and IGK and two consecutive rearrangements between diversity (D) and J genes, then between V and partially rearranged D-J genes for IGH [8]. After transcription, the V-(D)-J sequence is spliced to the constant (C) gene to give the final transcript. These rearrangement mechanisms involve a considerable repertoire of genes, which, combined with junctional N-diversity occurring during V-(D)-J gene recombination and with somatic mutations in B cell differentiation, result in a huge IG diversity [1,2,8].

The anatomical and physiological similarities between humans and animals have led researchers to investigate a large range of mechanisms and evaluate new therapies in animal models before applying their discoveries to humans [8]. Closely related to humans with 93% of its genome, the rhesus monkey (*Macaca mulatta*) is therefore one of the most widely used nonhuman primate species in biomedical research [9]. The rhesus monkey has played a key role in the development of vaccines for decades, such as hepatitis [10], tuberculosis [11,12,13], and most recently in the development of vaccines against human immunodeficiency virus-1 (HIV-1) [14,15,16,17,18,19,20] and against SARS-CoV-2 [21,22].

IMGT^®^, the international ImMunoGeneTics information system^®^, http://www.imgt.org, (accessed on 11 November 2021) [23], is the global reference in immunogenetics and immunoinformatics [1]. IMGT^®^ is a high-quality integrated knowledge resource specialized in IG, TR, major histocompatibility (MH) of human and other vertebrate species, and in the immunoglobulin superfamily (IgSF), MH superfamily (MhSF), and related proteins of the immune system (RPI) of vertebrates and invertebrates. Thanks to the currently “representative genome” of the rhesus monkey published on NCBI database: Mmul_10 [24], IMGT^®^ performed the complete annotation of the three IGH, IGK, and IGL loci of this genome, and the corresponding IG germline data were integrated within IMGT^®^ databases, tools, and web resources, improving the amount of rhesus monkey IG germline data which until then were based on Mmul_051212 [25] and individual gene sequences. It should be noticed that structural variations have been shown, in particular in the IGH locus of human [26] and in rhesus monkey [14,27,28]. The Mmul_10 annotation will be used as model and reference in IMGT^®^ for the characterization and the description of the IG genetic diversity in other *Macaca mulatta* genomes assemblies. During this study, a comprehensive analysis of the V, D (for IGH), J, and C genes was performed to determine their position in each IG locus, to characterize the allele functionality and to display the V, D, and J genes conventional recombination signal (RS) sequences. Furthermore, considering the important role of non-coding DNA regions in eukaryotic gene transcription as well as the variety and range of polymorphisms that have already been observed and studied in the 5′ untranslated region (5′UTR) of IG [29,30], a comprehensive in silico analysis of the 5′UTR of *Macaca mulatta* was carried out. Although the reasons for these changes have not yet been clearly revealed, these polymorphisms certainly modify the secondary structure of the promoter, affecting the stability, localization, transcription, and interaction of UTR with RNA-binding proteins [29,31]. Thus, identifying the regulatory elements of the promoter provides a basis for comparative analyses among the genes intra as well as inter species.

## 2. Materials and Methods

The biocuration was performed manually assisted by internally developed tools IMGT/LIGMotif [32], NtiToVald [33] and IMGT/Automat [33] based on the IMGT-ONTOLOGY axioms and concepts: “IDENTIFICATION”, “DESCRIPTION”, “CLASSIFICATION”, “NUMEROTATION”, “LOCALIZATION”, “ORIENTATION”, and “OBTENTION” [34]. IMGT-ONTOLOGY includes the controlled vocabulary and annotation rules which are indispensable to ensure accuracy and consistency.

The IMGT^®^ biocuration pipeline for locus annotation has been described previously [35]. Each locus sequence was localized on the corresponding chromosome and subsequently extracted from NCBI assembly [36] Mmul_10 in GenBank format. The delimitation of the locus was performed through research of the “IMGT bornes”, which are coding genes (other than IG or TR) conserved among species, located upstream of the first or downstream of the last gene of an IG or TR locus (http://www.imgt.org/IMGTindex/IMGTborne.php, (accessed on 11 November 2021)). The IMGT 5′ borne of the IGK locus is the paired box 8 (PAX8, Gene ID: 701906) gene and the IMGT 3′ borne of the locus is the ribose 5-phosphate isomerase A (RPIA, Gene ID: 699694) gene. The IMGT 5′ borne of the IGL locus is the DNA topoisomerase III (TOP3B, Gene ID: 698317) gene and the IMGT 3′ borne of the locus is the radial spoke head 14 homolog (RSPH14, Gene ID: 706814) gene. Similar to the *Homo sapiens* locus, the IMGT 5′ and IMGT 3′ bornes of the *Macaca mulatta* IGH locus could not be identified, therefore, the sequences of the V genes and C genes of the *Homo sapiens* IGH locus were used to localize the V-D-J-C-CLUSTER on the rhesus monkey genome assembly. The locus orientation on a chromosome can be either forward (FWD) or reverse (REV). Therefore, the REV locus sequences were placed in the 5′ to 3′ locus orientation. Each locus sequence thus obtained was assigned an IMGT^®^ accession number (IGL: IMGT000062, IGK: IMGT000063, IGH: IMGT000064).

According to the “CLASSIFICATION” axiom of IMGT-ONTOLOGY, the nomenclature of all V genes of each IG locus, was characterized based on the human V genes by using IMGT/V-QUEST [37] and NGPhylogeny.fr [38] to define the subgroups. All the V genes are designated by a number for the subgroup, followed by a hyphen and a number for their localization from 3′ to 5′ in the locus [2]. Two genes are assigned to the same subgroup if their V-REGION show a percentage of identity greater than 75% at the nucleotide level. The V genes which were pseudogenes and did not match in any subgroup were assigned to a clan also characterized according to the human clans (http://www.imgt.org/IMGTindex/Clan.php, (accessed on 11 November 2021)). Duplicated genes share the same name with an additional ‘D’ at the end of the second occurring gene. The nomenclature of the J genes and of the IGHD genes comprises a number for the sets defined according to the human sets [2], while the number corresponding to the localization is increased from 5′ to 3′ within the locus. Finally, C genes are designated according to their isotype, followed by a number if there was more than one gene, a number which also increased from 5′ to 3′ in the locus. An allele is a polymorphic variant of a gene which is characterized by mutations at the nucleotide level, in its core sequence (V-REGION, D-REGION, J-REGION, and C-REGION for V, D, J, and C genes, respectively). Alleles are designated by the gene name followed by an asterisk and a number with two digits starting from 01. The identification of an allelic polymorphism is performed by comparison of the IMGT reference sequence with a newly annotated genomic sequence and relies on the following rule: for a given mapped gene with the same IMGT position, the same IMGT allele name is assigned if the two core nucleotide sequences have 100% identity and the new genomic sequence is qualified as “sequence from the literature” for that allele. In case of less than 100% identity in the core, a new IMGT allele name is assigned, and the new genomic sequence becomes the “IMGT reference sequence”. The IMGT^®^ reference directories comprise the reference sequences of all gene alleles.

The functionality of the genes and alleles was defined according to the IMGT ‘functionality’ concept, more precisely the ‘IDENTIFICATION’ axiom of IMGT-ONTOLOGY, described in http://www.imgt.org/IMGTScientificChart/SequenceDescription/IMGTfunctionality.html (accessed on 11 November 2021). An allele is considered as functional (F) if its coding region has an open reading frame without stop codons, no defect in the splicing sites, recombination signals and/or regulatory elements. An allele is considered as open reading frame (ORF) if its coding region has an open reading frame without stop codons but shows alterations in the splicing sites, recombination signals, regulatory elements, and/or changes of conserved amino acids. A gene allele is considered as pseudogene (P) if the coding region has stop codon(s) and/or frameshift mutation(s).

The main concepts of the ‘DESCRIPTION’ axiom of IMGT-ONTOLOGY correspond to IMGT^®^ standardized labels in the tools and databases used to describe the organization of the IG genes in the IGH, IGL, and IGK loci [34]. The standardized annotation of nucleotide sequences (IMGT reference sequences and sequences from literature) is performed using IMGT^®^labels (http://www.imgt.org/ligmdb/label#, (accessed on 11 November 2021)) and integrated in IMGT/LIGM-DB [39]. This allows data entry of genes and alleles in IMGT/GENE-DB [40] and in the IMGT^®^ reference directory; and the entry of amino acid sequences in IMGT/3Dstructure-DB and IMGT/2Dstructure-DB [41]. IMGT^®^ reference directories are also used in the sequence analysis tools (IMGT/V-QUEST [37], IMGT/HighV-QUEST [42], and IMGT/DomainGapAlign [43]). The synthesis of the annotation of genomic data is integrated in dedicated sections of IMGT^®^ web resources: Locus representation, Locus description, Locus in genome assembly, Locus gene order, Locus Borne, Gene tables, Potential germline repertoire, Protein displays, Alignments of alleles, Colliers de Perles [44,45], and germline [CDR1-IMGT.CDR2-IMGT.CDR3-IMGT] lengths (http://imgt.org/IMGTrepertoire/, accessed on 11 November 2021).

The standardized annotation of nucleotide sequences (IMGT reference sequence and sequence from literature) is performed using IMGT^®^labels (http://www.imgt.org/ligmdb/label#, accessed on 11 November 2021) and integrated in IMGT/LIGM-DB [39]. This allows data entry of genes and alleles in IMGT/GENE-DB [40] and in the IMGT^®^ reference directory, and of amino acid sequences in IMGT/3Dstructure-DB and IMGT/2Dstructure-DB [41].

After integration of data in IMGT/GENE-DB, the 5′ UTR of the IG V genes and alleles were extracted. Those sequences were trimmed up to ~500 bp upstream the initiation codon (atg), which include all of the core promoter elements, as reported in the literature regarding *Homo sapiens* [46,47,48,49,50,51,52,53]. Then, a progressive multiple sequence alignment (MSA) was performed for the V genes 5′UTR of each locus and each subgroup separately. The MSA analysis was performed using MATLAB bioinformatics toolbox [54], as previously described in several studies [55,56], along with Clustal Omega tool/EMBL-EBI [57], MAFFT version 7 [58] and NGPhylogeny.fr [59]. The results of the MSA analysis were visualized through the Jalview platform [60]. Thus, guided by the IMGT^®^ reference of the *Homo sapiens* promoter sequences and distances between elements, the motif identification and extraction, along with the calculation of the distance between elements were carried out in *Macaca mulatta* loci. For further investigation and element validation, the study was assisted by bioinformatics tools and biological databases concerning eukaryotic promoter elements, including PROMO [61], gene-regulation [62], TRANSFAC database [63], Sequence Manipulation Suite [64], Transcription factor Affinity Prediction (TRAP) Web Tools [65], GPMiner [66], and SoftBerry/Nsite [67].

## 3. Results

### 3.1. Macaca mulatta IGH Locus

#### 3.1.1. Overview of the Locus

##### Genomic Organization of IGH Locus

**Figure 1:**
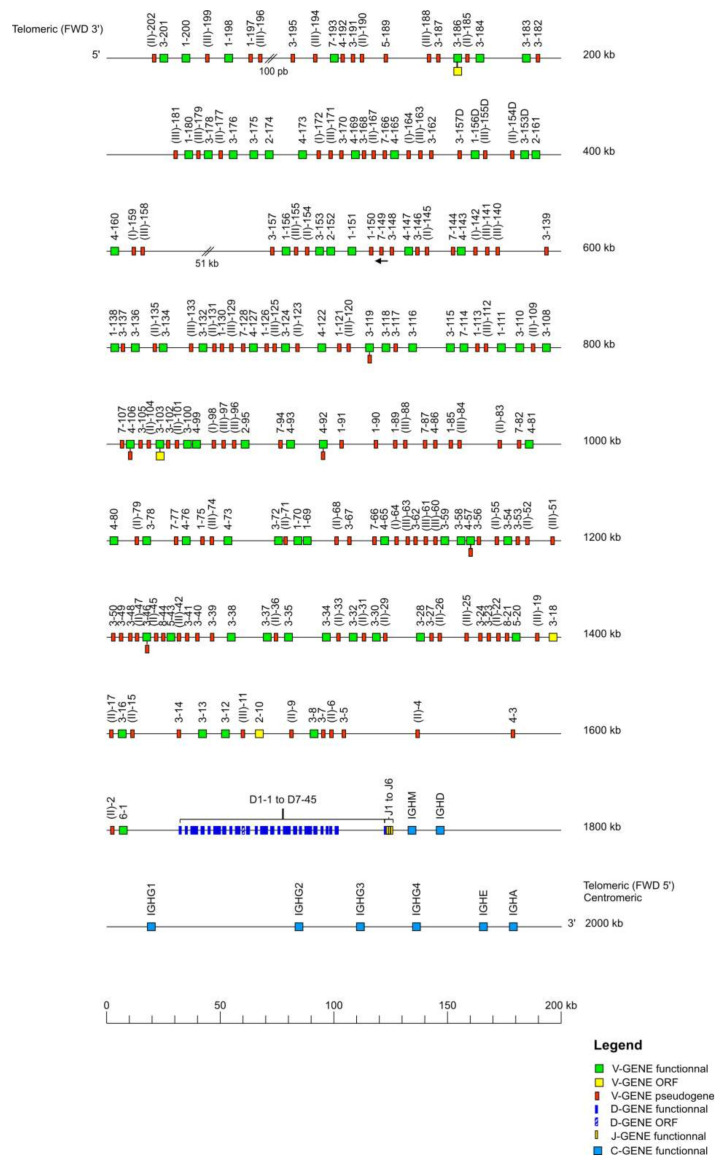
Locus representation of the *Macaca mulatta* (rhesus monkey) IGH deduced from the genome assembly Mmul_10. Reproduced with permission from IMGT^®^, the international ImMunoGeneTics information system^®^, http://www.imgt.org (accessed on 11 November 2021) The diagram shows the IGH genes and positions on the locus according to the IMGT nomenclature [1]. The arrows indicate an inverse transcriptional orientation in the locus. The V-D-J-C-CLUSTER is composed of IGHV(V6-1 to V(II)-202)-IGHD(D1-1 to D7-45)-IGHJ(J1 to J6)-IGHC(IGHM-IGHD-IGHG1-IGHG2-IGHG3-IGHG4-IGHE-IGHA). The 20 unlocalized V-GENE have a provisional nomenclature and are not present on the locus representation. Data available in IMGT Repertoire (IG and TR) http://www.imgt.org/IMGTrepertoire/, accessed on 11 November 2021 > Locus and genes > Locus representations > IGH > Rhesus monkey.

The *Macaca mulatta* (rhesus monkey) IGH locus is localized on chromosome 7 from position 167,900,000 to 169,868,564 in CM014342.1, Mmul_10. The orientation of the locus on the chromosome is REV and it spans 1969 kilobases (kb) (Table 1). The locus representation in Figure 1 encompasses 2000 kb from the most 5′ V gene IGHV(II)-202 (P) to the most 3′ C gene IGHA (F).

The IGH locus consists of 228 IGHV genes, among these genes 208 are localized on the IMGT^®^ reference sequence (IMGT000064) whereas 20 unlocalized genes come from previous annotated sequences. The 228 IGHV genes of the rhesus monkey belong to eight subgroups and three clans. The 45 IGHD genes belong to seven IGHD sets. The seven IGHJ genes belong to six IGHJ sets, and the eight IGHC genes belong to eight isotypes (Table 1). The IGHV genes span 1600 kb, the IGHD genes span 103 kb, the IGHJ genes span 6 kb, and the IGHC genes span 260 kb. The IMGT^®^ reference sequence (IMGT000064) has two gaps from position 48,631 to 48,730 and from position 398,207 to 449,403.

##### Characterization of the IGH Genes

Briefly, 288 genes and 447 alleles of the IGH locus have been annotated and integrated in the IMGT^®^ databases. Among the 288 genes, we have identified for the IGHV: 79 F, two ORF, 140 P, and seven genes that have alleles with different functionalities (F or ORF ‘FO’: IGHV3-186, IGHV3-103; F or P ‘FP’: IGHV3-46, IGHV3-119, IGHV4-57, IGHV4-92 and IGHV4-106) (Table 2); for the IGHD: 44 F and one ORF; for the IGHJ: seven F, and for the IGHC eight F. Six duplicated V genes were found within the IGH locus: IGHV(II)-33D (P), IGHV3-153D (F), IGHV(II)-154D (P), IGHV(III)-155D (P), IGHV1-156D (F), and IGHV3-157D (P).

IGHV3 is the most represented subgroup [68] with 78 genes and 124 alleles. It also presents the greatest number of F with 39 genes and 62 alleles, followed by the IGHV1 and IGHV4 subgroups with 11 F and 19 F genes respectively. The IGHV7 subgroup has 14 genes however most are P (12 P) whereas the IGHV2 subgroup has fewer genes (seven) than IGHV7 but only one is P. All genes having alleles with different functionalities belong to the subgroups IGHV3 (two FO, two FP) and IGHV4 (three FP). All genes of the clans are P by definition. The IGHV(II) and IGHV(III) are the most represented clans with 35 and 32 genes, respectively, while IGHV(I) has six genes (Table 3).

Each IGHD set comprises seven genes except for the IGHD1 set which is the most represented with nine genes and IGHD7 set which is the least represented with one gene. All IGHD genes are F except for one gene (IGHD5-18) which is ORF (Table 4). The IGHJ5 set comprises two genes (IGHJ5-1 and IGHJ5-2), while the remaining IGHJ sets comprise a single gene. All IGHJ genes are F (Table 5). For the IGHC genes, four genes belong to the IGHG (IGHG1, IGHG2, IGHG3 and IGHG4) while the other four genes encode the isotypes IGHA, IGHD, IGHE and IGHM, respectively. The IGHC genes are F except for the IGHM and IGHG3 gene which have alleles with two different functionalities (F or P). In fact, both have one allele P, while the other two IGHM and six IGHG3 alleles are F (Table 6).

Finally, the IMGT^®^ databases count, for the IGHV, 228 genes and 336 alleles; for the IGHD, 45 genes and 49 alleles; for the IGHJ, seven genes and 10 alleles and for the IGHC eight genes and 52 alleles. Further, 95 additional (26 F, 69 P) IGHV genes have been annotated from the IMGT^®^ reference sequence IMGT000064 (Mmul_10) compared to the IGH locus sequences in assembly Mmul_051212. However, two IGHD genes (IGHD1-1-1 and IGHD4-41) were only found in the scaffolds of the assembly Mmul_051212 with accession numbers NW_001121239 and NW_001121238 respectively.

#### 3.1.2. CDR-IMGT Distributions and IMGT Proteins Displays

The three largest subgroups, IGHV1, IGHV3, and IGHV4, have several different CDR-IMGT lengths. However, there are some CDR-IMGT lengths that are more frequently represented than others: [8.8.2] for IGHV1 with 14 genes (11 F, three in-frame P), for IGHV3 with 22 genes (17 F, five in-frame P), for IGHV4 with eight genes (eight F), for IGHV5 with three genes (two F, one in-frame P) and IGHV7 with nine genes (two F, seven in-frame P); [8.10.2] for IGHV3 with 15 genes (12 F, one ORF, two in-frame P). The CDR-IMGT lengths [8.8.2] are found in almost every subgroup except in IGHV2 and IGHV6 subgroups (Table 7) [69]. The IGHV8 subgroup is not shown in the table because all its genes are out-of-frame P.

**Figure 2:**
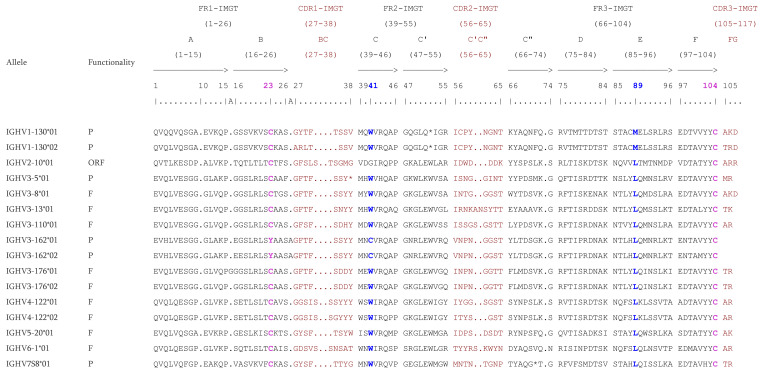
IMGT Protein display of the *Macaca mulatta* (rhesus monkey) IGHV genes. Only a few functional genes, ORF and in-frame P are shown as examples. The outline of the CDR-IMGT and FR-IMGT are according to the IMGT unique numbering for V-REGION [70]. The four conserved amino acids are shaded in pink for the C23 and C104, blue for the W41 and hydrophobic AA 89. * indicates in frame STOP-CODON. The CDR-IMGT are shaded in maroon while the FR-IMGT are in black. Data available in IMGT Repertoire (IG and TR) http://www.imgt.org/IMGTrepertoire/, accessed on 11 November 2021 > Proteins and alleles > Protein displays > V-DOMAIN > IGHV > Rhesus monkey (*Macaca mulatta*) or IMGT DomainDisplay http://www.imgt.org/3Dstructure-DB/cgi/DomainDisplay.cgi, accessed on 11 November 2021.

The protein displays of some IGHV genes are presented with examples of CDR-IMGT lengths (Figure 2). There are cases where a gene has alleles with different CDR-IMGT lengths, the genes IGHV1-130 and IGHV4-122 being examples of that. The allele IGHV1-130*01 has [8.8.3] as CDR-IMGT lengths, whereas the allele IGHV1-130*02 has [7.8.3]. The allele IGHV4-122*01 has [9.8.2] and the CDR-IMGT lengths [9.7.2] is found on the allele IGHV4-122*02. This is due to a deletion of an amino acid (AA) in the CDR but there are also cases of insertion, for example the allele IGHV3-176*01 which has an additional position (15A) according to the IMGT unique numbering [70] or the allele IGHV3-162*01 and IGHV3-162*02 which have an insertion (A) at position 26A. The four conserved AA are highlighted: the two cysteines at positions 23 and 104 (C23 and C104), the tryptophan at position 41 (W41), and a conserved hydrophobic AA at position 89 [70]. Most of the time, the hydrophobic AA is a leucine. However, for the IGHV1 subgroup, it is methionine.

#### 3.1.3. RS Sequences

**Figure 3:**
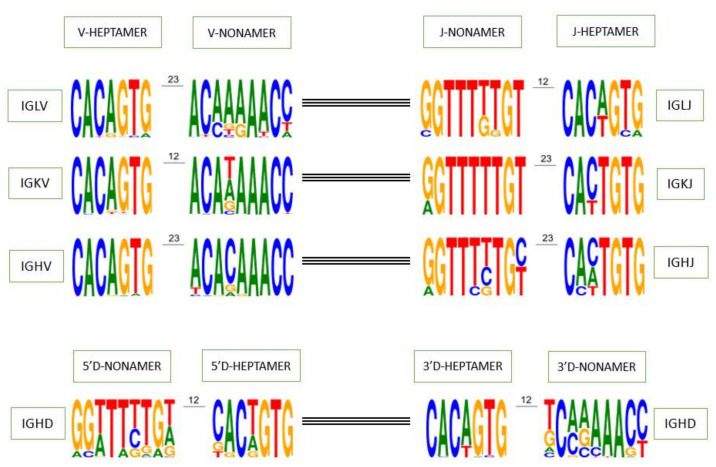
Recombination signal sequences of functional V, D and J genes (V-RS, 5′D-RS, 3′D-RS and J-RS respectively) for each IG locus of the rhesus monkey. The height of symbols indicates the relative frequency of each nucleotide at that position. Data extracted from IMGT Repertoire (IG and TR) http://www.imgt.org/genedb/, accessed on 11 November 2021 and depicted as sequence logos by WebLogo [71].

The V-HEPTAMER and V-NONAMER consensus sequences of all functional IGHV genes of the rhesus monkey are ‘cacagtg’ and ‘acacaaacc’ (Figure 3). ‘cacagtg’ is also the consensus sequence of all the IGHV subgroups of the rhesus monkey except for the IGHV2, which is instead ‘cacagag’ (Supplementary Table S1). The subgroups IGHV3, IGHV4, and IGHV6 share the same nonamer with the consensus sequence of all functional IGHV genes however the IGHV1, IGHV2 and IGHV5 subgroup have respectively ‘tcagaaacc’, ‘acaagaacc’, and ‘ccaaaaacc’ as their consensus sequences. The IGHV7 subgroup does not have a consensus sequence for the nonamer, the two functional genes of this subgroup (IGHV7-114 and IGHV7-193) represent a total of three alleles and each one has a different nonamer (Supplementary Table S1a). The J-HEPTAMER and J-NONAMER consensus sequence of all IGHJ functional genes are ‘cactgtg’ or ‘caatgtg’ and ‘ggtttttgt’ respectively (Figure 3). These J-HEPTAMER are observed on the IGHJ1, IGHJ4 and IGHJ5, whereas this J-NONAMER is observed on the IGHJ4 and IGHJ6 (Supplementary Table S1b). The 5′D-HEPTAMER and 5′D-NONAMER consensus sequence of IGHD functional genes are ‘cactgtg’ and ‘ggtttttgt’. The former is the consensus sequence of the IGHD2 and IGHD7 sets while the latter is not the motif predominately found on any set (Supplementary Table S1c). The same pattern holds true for the 3′D-HEPTAMER and 3′D-NONAMER. The consensus sequences of IGHD functional genes are ‘cacagtg’ and ‘tcaaaaacc’. The motif ‘cacagtg’ is found as consensus sequence in all sets except for the IGHD1, while the motif ‘tcaaaaacc’ is only found in the IGHD3 set (Supplementary Table S1).

#### 3.1.4. 5′ UTR Analysis of the IGHV Subgroup

**Figure 4:**
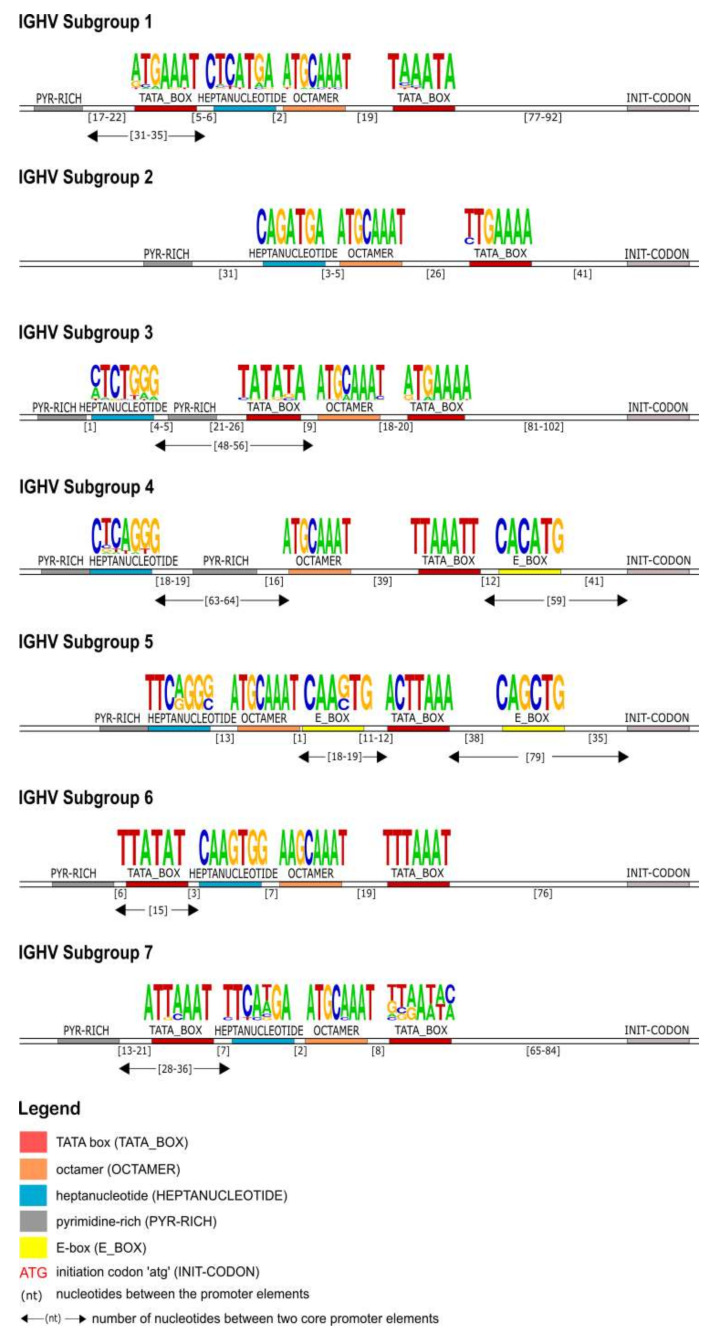
Schematic representation of the IGHV genes promoter organization per subgroup, based on the regulatory elements consensus sequences, positions and distances. Each element is represented by a specific color according to the IMGT color menu for the organization of the V-GENE promoters. The IMGT labels of each element are mentioned in parentheses in the legend.

A highly conserved octamer motif is identified upstream of the initiation codon (ATG) in all of the IGHV subgroups. Except for the subgroup IGHV6, the octamer consensus sequence found in all other subgroups was 5′-ATGCAAAT-3′. The TATA box is located between the ATG and octamer in all of the IGHV gene promoters and its sequence is characteristic for each subgroup. Upstream of the octamer, an heptanucleotide motif and a pyrimidine-rich region were found in every subgroup. The distance between these two elements and the length of the pyrimidine-rich region are representative of each subgroup. Additional elements are found in the promoter region of some subgroups. Subgroups IGHV3 and IGHV4 have an additional pyrimidine-rich region between the octamer and the heptanucleotide. Two E-box motifs are observed between the core elements in subgroups IGHV4 and IGHV5. Finally, in IGHV1, IGHV6, and IGHV7, an additional TATA box is located between the pyrimidine-rich region and the heptanucleotide, and for IGHV3, it was found between the heptanucleotide and the octamer (Figure 4).

### 3.2. Macaca mulatta IGL Locus

#### 3.2.1. Overview of the Locus

##### Genomic Organization of IGL Locus

**Figure 5:**
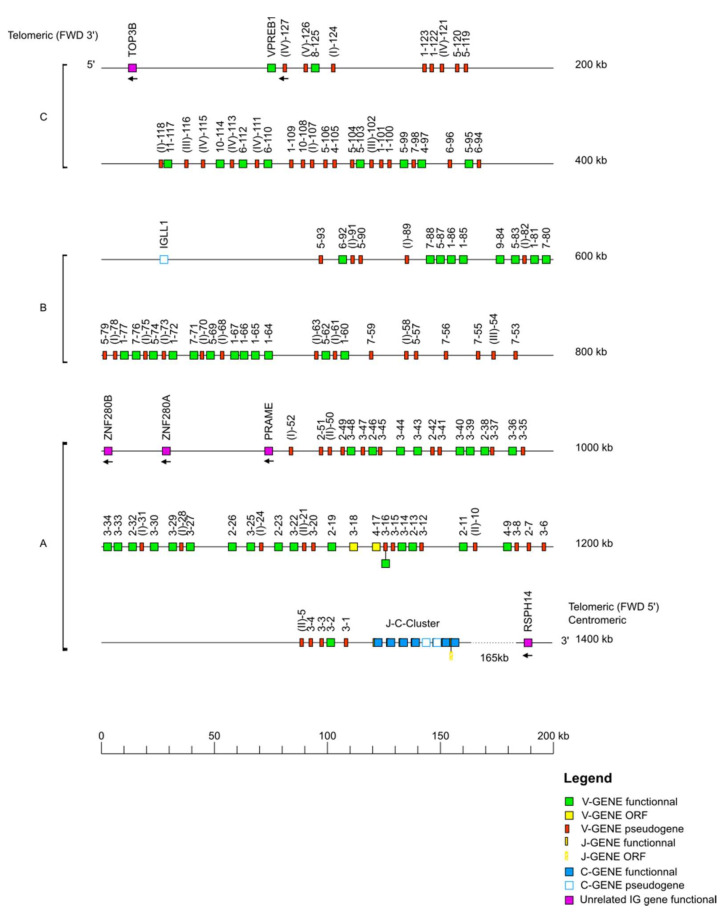
Locus representation of the *Macaca mulatta* (rhesus monkey) IGL locus deduced from the genome assembly Mmul_10. Reproduced with permission from IMGT^®^, the international ImMunoGeneTics information system^®^, http://www.imgt.org, (accessed on 11 November 2021). A dotted line… indicates the distance in kb between the locus and the IMGT 3′ borne. A, B, C refer to three distinct V-CLUSTER based on the IGLV gene subgroup content, by homology with the *Homo sapiens* IGL locus. The IGL J-C-CLUSTER comprises 8 cassettes indicated by the numbers 1 to 7 (IGLJ1-IGLC1, IGLJ2-IGLC2, IGLJ2A-IGLC2A, IGLJ3-IGLC3, IGLJ4-IGLC4, IGLJ5-IGLC5, IGLJ6-IGLC6 and IGLJ7-IGLC7 respectively) (Supplementary Figure S1). The 22 unlocalized V-GENE have a provisional nomenclature and are not present on the locus representation. Data available in IMGT Repertoire (IG and TR) http://www.imgt.org/IMGTrepertoire/, accessed on 11 November 2021 > Locus and genes > Locus representations > IGL > Rhesus monkey.

The *Macaca mulatta* (rhesus monkey) IGL locus is located on chromosome 10 from position 29,621,424 to 30,922,134 in CM014345.1, Mmul_10 and the orientation of the locus on the chromosome is REV. The IGL locus spans 1301 kb from 10 kb upstream of the most 5′ gene (non-IG VPREB1 (F)), to 10 kb downstream of the most 3′ gene (IGLC7 (F)). The locus representation (Figure 5) encompasses 1400 kb including the IMGT 5′ borne (TOP3B) and the IMGT 3′ borne (RSPH14).

The rhesus monkey IGL locus consists of 149 IGLV genes (127 genes localized on the locus + 22 unlocalized) belonging to 11 subgroups and five clans, eight IGLJ genes belonging to eight IGLJ sets and eight IGLC genes (Table 1) [72]. The IGLV genes span 1247 kb, whereas the IGLJ genes and IGLC genes span 26 kb and 28 kb, respectively. TOP3B has been identified 48 kb upstream of VPREB1 and RSPH14 has been identified 165 kb downstream of IGLC7.

The rhesus monkey IGL locus has three distinct V-CLUSTER A, B, and C based on the IGLV gene subgroup content, by comparison with the *Homo sapiens* IGL locus. Within the V-CLUSTER A, there are three functional non-IG genes (PRAME, ZNF280A and ZNF280B) and on the V-CLUSTER B there is an IGLL1 (P).

##### Characterization of the IGL Genes

Briefly, 165 genes and 247 alleles have been annotated and integrated on the IMGT^®^ databases. Among the 165 genes, we have identified: for the IGLV, 72 F, two ORF, 74 P, and one gene (IGLV3-16), which has two alleles with different functionalities (F or P) (Table 2); for the IGLJ: nine F, one ORF and one gene (IGLJ7) which also has two alleles with different functionalities (F or ORF); and for the IGLC: six F and two P.

The most represented subgroup in the IGL locus is IGLV3 with 25 F genes and 35 F alleles, one gene and three alleles have been identified as ORF, 16 genes and 23 alleles have been identified as P, and one gene has a double functionality (FP) (Table 8). The IGLV1, IGLV2, and IGLV5 are also well represented with 14 F genes, 11 F genes, and eight F genes, respectively. In contrast, the least represented subgroups are IGLV8, IGLV9, IGLV10, and IGLV11 with only one F gene. In addition to the 11 subgroups, the IGL locus also has five clans (IGLV(I), IGLV(II), IGLV(III), IGLV(IV), and IGLV(V)), all pseudogenes per definition. The most represented clan is IGLV(I) with 18 genes and 24 alleles, whereas the IGLV(V) has only one gene and allele.

The J-C-CLUSTER comprises eight IGLJ-IGLC cassettes indicated by the numbers 1–7 (IGLJ1-IGLC1, IGLJ2-IGLC2, IGLJ2A-IGLC2A, IGLJ3-IGLC3, IGLJ4-IGLC4, IGLJ5-IGLC5, IGLJ6-IGLC6, and IGLJ7-IGLC7, respectively) (Supplementary Figure S1). The IGLJ4 gene and one allele of the IGLJ7 are ORF, whereas all other IGLJ genes are F (Table 9). Six IGLC genes and their alleles are F, and the other, IGLC4 and IGLC5, are P (Table 10).

Finally, the IMGT^®^ databases count 149 IGLV genes and 225 alleles among which 72 genes and 108 alleles are F, two genes and five alleles are ORF, 74 genes and 110 alleles are P and one gene and two alleles have two functionalities (FP); eight genes and nine alleles for the IGLJ and eight genes and 13 alleles for the IGLC. Further, 27 additional genes were annotated from the IMGT reference sequence IMGT000062 (Mmul_10) compared to the IGL locus sequence NW_001095158 (Mmul_051212 assembly) which has 245 gaps. Among the 27 genes, 17 IGLV genes were P and eight IGLV, one IGLJ, and one IGLC were F.

#### 3.2.2. CDR-IMGT Distributions and IMGT Proteins Displays

The CDR-IMGT length is well conserved within the subgroup IGLV2. All the genes within this subgroup (11 F and one in-frame P) have the same CDR-IMGT lengths [9.3.9]. The same conservation is observed for the subgroups IGLV6 (3 F) and IGLV7 (4 F and 3 in-frame P) which have respectively the CDR-IMGT lengths [8.3.8] and [9.3.8]. The IGLV1, IGLV4 and IGLV5 have two different CDR-IMGT lengths within their subgroups. The most frequently found CDR-IMGT lengths in IGLV1 and IGLV5 are respectively [8.3.9] (12 F) and [9.7.8] (seven F and one in-frame P); for IGLV4, the two different CDR-IMGT lengths ([7.7.7] and [7.7.12]) are both observed in two genes. The IGLV3 subgroup has four different CDR-IMGT lengths ([6.3.7], [6.3.8], [6.3.9], and [6.3.12]), but the most frequent one is [6.3.9] with 18 F, one ORF, and one in-frame P. The subgroups IGLV8, IGLV9, IGLV10, and IGLV11 comprise a single gene and their CDR-IMGT lengths are [9.3.8], [7.8.12], [8.3.9], and [9.7.8], respectively (Table 11).

**Figure 6:**
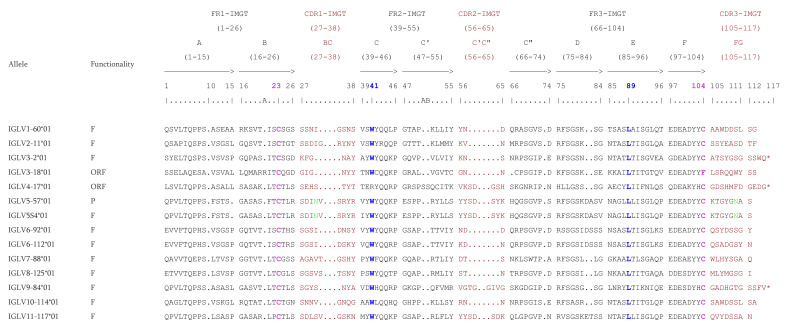
IMGT Protein display of the *Macaca mulatta* (rhesus monkey) IGLV genes. Only a few functional genes, ORF and in-frame P are shown, as examples. The outline of the CDR-IMGT and FR-IMGT are according to the IMGT unique numbering for V-REGION [70]. The four conserved amino acids are shaded in pink for the C23 and C104, blue for the W41 and hydrophobic AA 89. * indicates in frame STOP-CODON. The CDR-IMGT are shaded in maroon while the FR-IMGT are in black. Data available in IMGT Repertoire (IG and TR) http://www.imgt.org/IMGTrepertoire/, accessed on 11 November 2021 > Proteins and alleles > Protein displays > V-DOMAIN > IGLV > Rhesus monkey (*Macaca mulatta*) or IMGT DomainDisplay http://www.imgt.org/3Dstructure-DB/cgi/DomainDisplay.cgi, accessed on 11 November 2021.

The IGLV genes have a long CDR3-IMGT which varies from 7 to 12 AA. The four conserved AA (C23, W41, hydrophobic 89 and C104) are present in all functional IGLV genes. Only one or two examples for each subgroup is shown on Figure 6. Whether it is the F, ORF, or in-frame P, all IGLV genes count 25 AA in the FR1-IMGT with a gap at position 10 according to the IMGT unique numbering [70], however there are exception like the allele IGLV3-18*01 which has an insertion of 1 AA (R) at position 20A or the alleles IGLV5-57*01 and IGLV5S4*01 which have a deletion of 1 AA at position 15. The IGLV genes of rhesus monkey count 17 AA in the FR2-IMGT (from position 39 to 55) but the allele IGLV4-17*01, which is ORF because of the non-conserved W41, has an insertion of 2 AA (SS) at positions 50A and 50B. The subgroups IGLV5, IGLV6 and the only representative of subgroup 11 (IGLV11-117) have a complete D-STRAND (75–84) of the FR3-IMGT.

#### 3.2.3. RS Sequences

The V-HEPTAMER and V-NONAMER consensus sequences of all IGLV functional genes are ‘cacagtg’ and ‘acaaaaacc’ (Figure 3), however they may be distinct for particular subgroups: the consensus sequence of the V-HEPTAMER of IGLV6 subgroup is ‘cacagta’, and no consensus sequence is defined for IGLV8 subgroup since the two alleles of the single gene IGLV8-125 have two different V-HEPTAMER. Concerning the V-NONAMER, only IGLV5, IGLV9, and IGLV11 share the IGLV ‘acaaaaacc’ consensus sequence (Supplementary Table S2a).

The J-HEPTAMER and J-NONAMER consensus sequences of all IGLJ functional genes are ‘cacagtg’ and ‘ggtttttgt’ (Figure 3). However, they may be distinct for particular sets: IGLJ1 and IGLJ7 have the same J-HEPTAMER ‘cactgtg’ while the IGLJ5 has two different nucleotides ‘cacagca’. Concerning the J-NONAMER, only IGLJ2, IGLJ2A, and IGLJ5, share IGLJ nonamer consensus sequence (Supplementary Table S2b).

#### 3.2.4. 5′ UTR Analysis of the IGLV Subgroup

**Figure 7:**
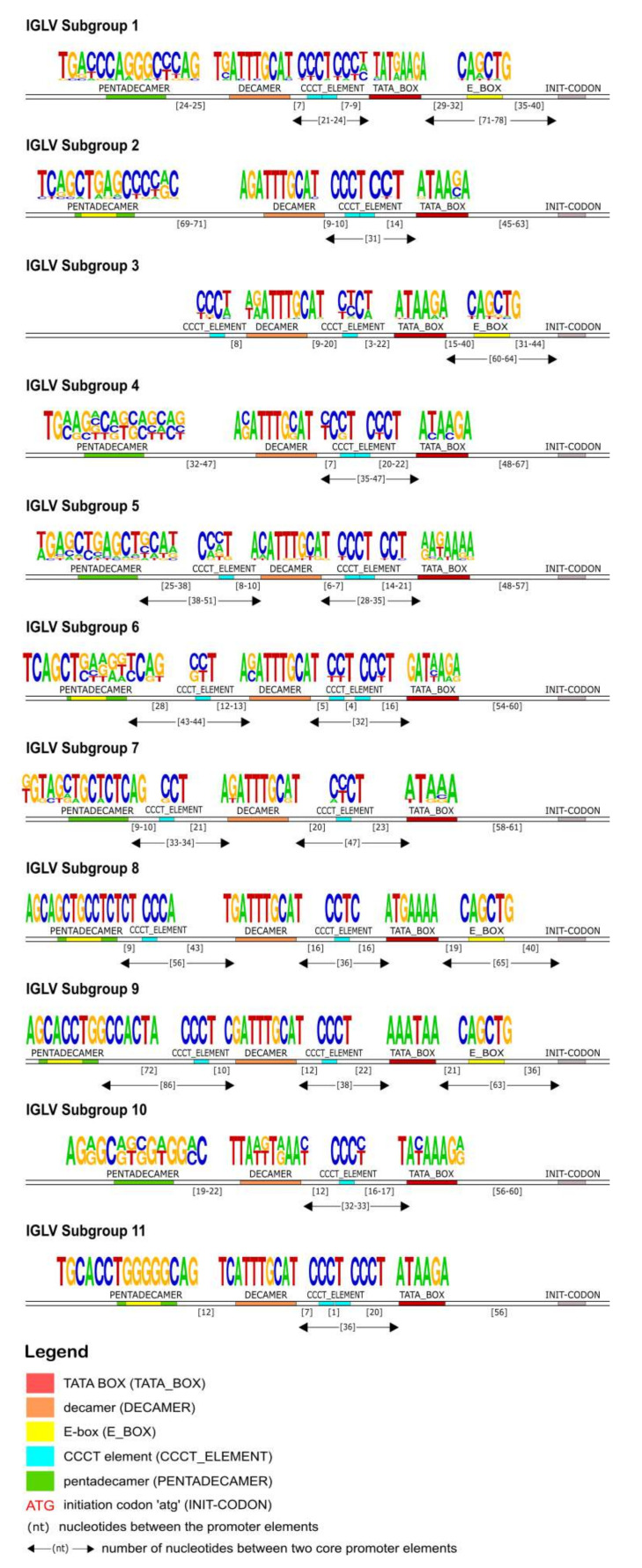
Schematic representation of the IGLV genes promoter organization per subgroup, based on the regulatory elements consensus sequences, positions and distances. Each element is represented by a specific color according to the IMGT color menu for the organization of the V-GENE promoters. The IMGT labels of each element are mentioned in parentheses in the legend.

In the 5′UTR of all IGLV subgroups, a TATA box was detected approximately 60 nucleotides upstream of the initiation codon (ATG) (Figure 7). Upstream of the TATA box, a highly conserved decamer element was identified in all subgroups and its consensus sequence was calculated as 5′-AGATTTGCAT-3′. A pentadecamer element was observed upstream of the decamer in all subgroups except for the IGLV3 subgroup. The position of the pentadecamer, as well as its sequence, varied among the genes of different Subgroups in IGLV promoters. However, a noticeable conservation of this element can be observed in the genes of the same subgroup, thus its position and consensus sequence are distinctive and specific for every subgroup. A CCCT element was located between the pentadecamer and the decamer for the majority of subgroups. One or two repeats of the CCCT element were also detected between the decamer and the TATA box in all IGLV subgroups. Finally, an E-box motif (5′ CAnnTG 3′) was found within the pentadecamer (for subgroups IGLV2, IGLV6, IGLV8, IGLV9, and IGLV11) and/or between the TATA box and ATG (for subgroups IGLV1, IGLV3, and IGLV9).

### 3.3. Macaca mulatta IGK Locus

#### 3.3.1. Overview of the Locus

##### Genomic Organization of IGK Locus

The *Macaca mulatta* (rhesus monkey) IGK locus is localized on chromosome 13 from position 16,784,193 to 18,140,859 in CM014348.1, Mmul_10 and the orientation of the locus on the chromosome is FWD. The locus spans 1357 kb, from 10 kb upstream of the most 5′ gene in the locus IGKV2-105 (P), to 10 kb downstream of the most 3′ gene in the locus IGKC (F).

**Figure 8:**
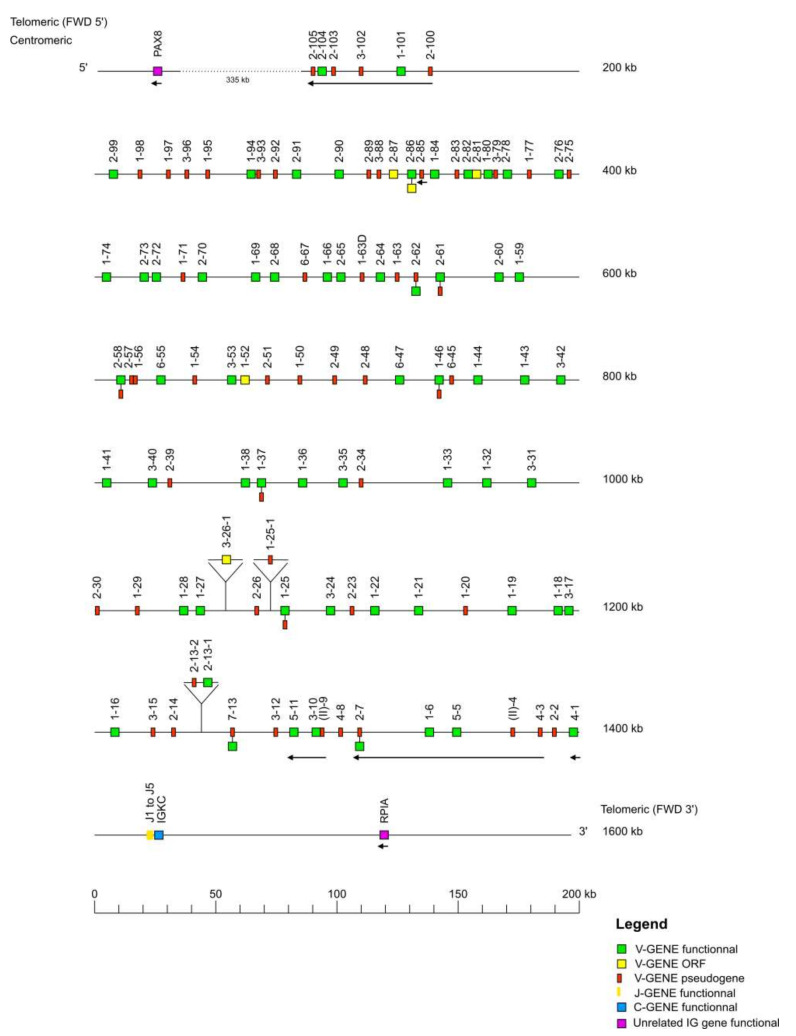
Locus representation of the *Macaca mulatta* (rhesus monkey) IGK deduced from the genome assembly Mmul_10. Reproduced with permission from IMGT^®^, the international ImMunoGeneTics information system^®^, http://www.imgt.org (accessed on 11 November 2021). A dotted line… indicates the distance in kb between the locus and the IMGT 5′ borne. These distances are not represented at scale and are not included in the numbers displayed at the right ends of these two lines. IGKV2-13-1, IGKV2-13-2, IGKV1-25-1 and IGKV3-26-1 genes are not found in the Mmul_10 assembly (IMGT000063 accession number) but found in the accession number NW_001099007. Data available in IMGT Repertoire (IG and TR) http://www.imgt.org/IMGTrepertoire/, accessed on 11 November 2021 > Locus and genes > Locus representations > IGK > Rhesus monkey.

The locus representation (Figure 8) encompasses 1600 kb including the IMGT 5′ borne PAX8 identified 335 kb upstream of IGKV2-105 and the IMGT 3′ borne RPIA identified 94 kb downstream of IGKC. The IGK locus consists of 138 IGKV genes (110 genes localized on the locus + 28 unlocalized) belonging to seven subgroups and one clan, five IGKJ genes, and one IGKC gene (Table 1). The IGKV genes span 1331 kb, whereas the IGKJ and IGKC genes span respectively 14 kb and 12 kb.

##### Characterization of the IGK Genes

Briefly, 144 genes and 214 alleles of the IGK locus have been annotated and integrated into the IMGT^®^ databases. Among the 144 genes, we have found for the IGKV: 76 F, six ORF, 47 P and nine genes which have alleles with different functionalities (Functional or ORF: IGKV2-86; Functional or pseudogene: IGKV1-25, IGKV1-37, IGKV1-46, IGKV2-7, IGKV2-58, IGKV2-61, IGKV2-62, IGKV7-13) (Table 2); for the IGKJ genes, five are F and the only IGKC gene is F.

The subgroups IGKV1 and IGKV2 are the most represented (≥50 genes for each subgroup) [73]. The IGKV1 subgroup has the greatest number of F genes with 39 genes out of 56, followed by the IGKV2 subgroup with 21 F out of 50 (Table 12). The IGKV3 subgroup has 11 F genes out of 19. In contrast, the least represented subgroups are IGKV4, IGKV5, IGKV6, and IGKV7 with respectively four, two, four, and one genes. The two genes of the IGKV5 subgroup are F. The IGKV7 subgroup comprises only one gene: IGKV7-13 which has two alleles with different functionalities (functional or pseudogene). In addition to the seven subgroups, the IGK locus also has one clan IGKV(II) which comprises two truncated pseudogenes.

Finally, the IMGT^®^ databases count 138 IGKV genes and 206 alleles. All the five J genes of the IGK locus are F. For now, one allele of each IGKJ gene has been annotated (Table 13). The only IGKC is F and currently two alleles have been described. Moreover, 43 additional (21 F, two ORF, 20 P) IGKV genes have been annotated from the IMGT^®^ reference sequence IMGT000063 (Mmul_10) compared to the IGK locus sequence NW_001099007 (Mmul_05121). However, four missing IGKV genes on the sequence IMGT000063 (IGKV2-13-1, IGKV2-13-2, IGKV1-25-1, and IGKV3-26-1) were found in the accession number NW_001099007.

#### 3.3.2. CDR Distributions & Proteins Displays

The CDR-IMGT length [6.3.7] is conserved within IGKV1 which is the most represented subgroup with 42 F, one ORF, and three in-frame P. This CDR-IMGT length is also observed in the subgroups IGKV3, IGKV5, and IGKV6, with 11 F, one ORF for the IGKV3, two F for IGKV5, and two F, one in-frame P for the IGKV6. The CDR2-IMGT length is the same for all genes of all subgroups, whereas the CDR1-IMGT length and CDR3-IMGT length are variable (Table 14).

**Figure 9:**
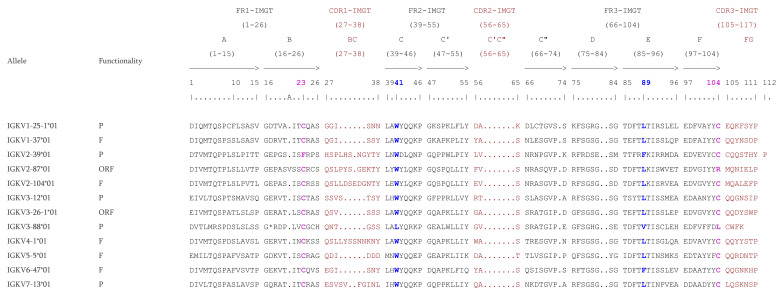
IMGT Protein display of the Rhesus monkey IGKV genes. Only a few functional genes, ORF and in-frame P are shown. The outline of complementarity determining regions (CDR-IMGT) and framework regions (FR-IMGT) are according to the IMGT unique numbering for V-REGION [70]. The four conserved amino acids are shaded in pink for the C23 and C104, blue for the W41 and hydrophobic AA 89. * indicates in frame STOP-CODON. The CDR-IMGT are shaded in maroon while the FR-IMGT are in black. Data available in IMGT Repertoire (IG and TR) http://www.imgt.org/IMGTrepertoire/, accessed on 11 November 2021 > Proteins and alleles > Protein displays > V-DOMAIN > IGKV > Rhesus monkey (*Macaca mulatta*) or IMGT DomainDisplay http://www.imgt.org/3Dstructure-DB/cgi/DomainDisplay.cgi, accessed on 11 November 2021.

The CDR1-IMGT and CDR3-IMGT lengths fluctuate from 6 to 12 AA and from 4 to 8 AA, respectively. The four conserved AA (C23, W41, hydrophobic 89 and C104) are present in all functional IGKV genes. However, due to their numbers, only a few genes are shown on Figure 9. In contrast, for ORF or P alleles shown on Figure 9, some conserved AA are replaced by another AA. For example, the allele IGKV2-87*01 (ORF) has an Arginine (R) instead of the C104; the allele IGKV3-88*01 (P) has a Leucine (L) instead of the W41 and C104; the IGKV2-39*01(P) has a Phenylalanine (F) instead of the C23. Whether they are F, ORF or in-frame P, the IGKV genes count 26 AA at FR1-IMGT. However, the allele IGKV2-87*01 has an insertion of 1 AA (V) at the position 20A on the FR1-IMGT. The IGKV genes of *Macaca mulatta* have no gaps in FR2-IMGT (17 AA from position 39 to 55) but for the FR3-IMGT there are gaps at positions 73, 81, and 82 according to the IMGT unique numbering [70].

#### 3.3.3. RS Sequences

The V-HEPTAMER and V-NONAMER consensus sequences of all IGKV functional genes are ‘cacagtg’ and ‘acaaaaacc’ (Figure 3). However, they may be distinct for particular subgroups: the consensus sequence of the V-HEPTAMER of IGKV6 subgroup is ‘cacactg’. Concerning the V-NONAMER, only IGKV5 and IGKV7 subgroups share the IGKV ‘acaaaaacc’ consensus sequence (Supplementary Table S3a).

The J-HEPTAMER and J-NONAMER consensus sequences of all IGKJ functional genes are ‘cactgtg’ and ‘ggtttttgt’ (Figure 3), hovewer the J-HEPTAMER and J-NONAMER of the IGKJ2 and IGKJ5 have one mutation compared to the other functional J genes (‘cattgtg’ and ‘agtttttgt’ for the IGKJ2; ‘tactgtg’ and ‘gatttttgt’ for the IGKJ5) (Supplementary Table S3b).

#### 3.3.4. 5′ UTR Analysis of the IGKV Subgroup

**Figure 10:**
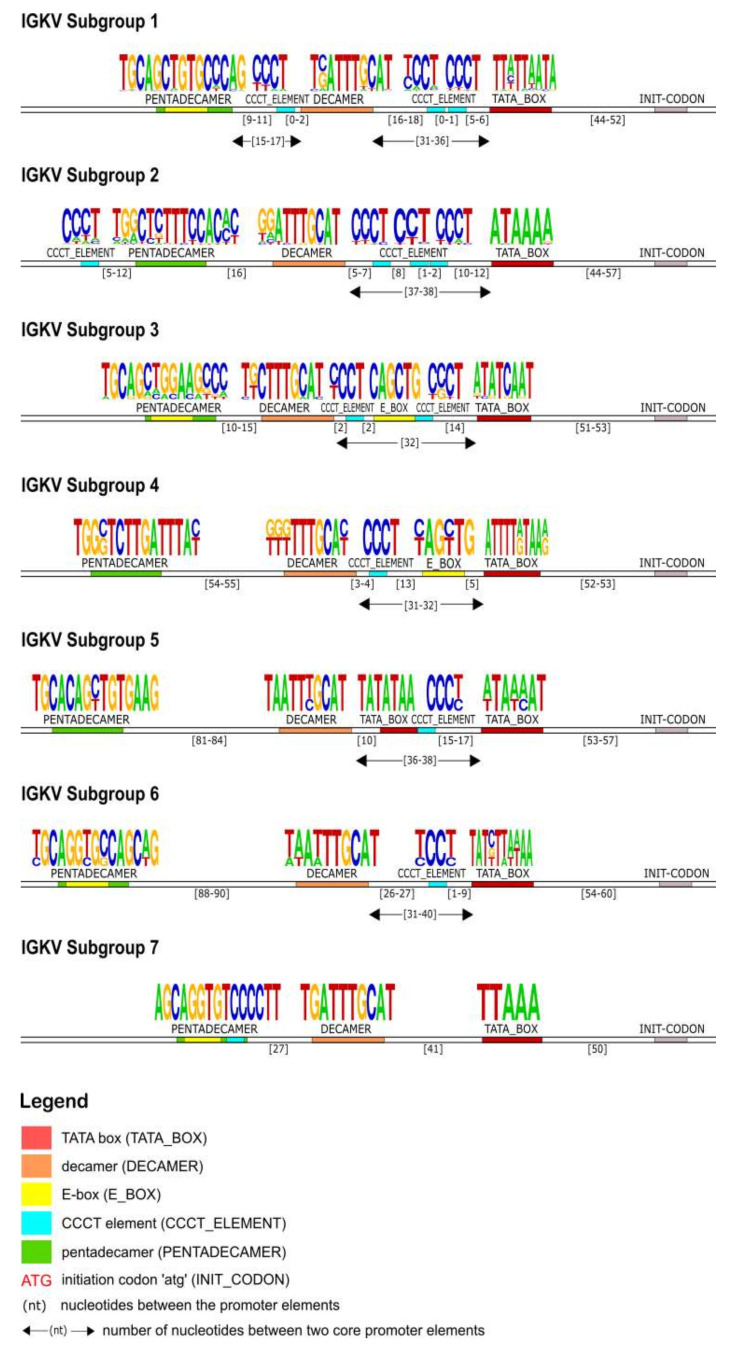
Schematic representation of the IGKV genes promoter organization per subgroup, based on the regulatory elements consensus sequences, positions and distances. Each element is represented by a specific color according to the IMGT color menu for the organization of the V-GENE promoters. The IMGT labels of each element are mentioned in parentheses in the legend.

In the 5′UTR of the IG kappa chain, right upstream of the initiation codon, the TATA box motif was identified first in all of the subgroups. It was located on average 53 nucleotides upstream of the initiation codon (ATG) and it is composed of five to ten A/T repeats. Upstream of the TATA box, a CCCT element (TCCT for the IGKV5 subgroup) was observed in all subgroups except for IGKV7. The most conserved core promoter element was decamer (5′-nnATTTGCAT-3′) and it was located upstream of the previously mentioned core elements (TATA box and CCCT element). The pentadecamer motif was located within 11–90 nucleotides upstream of the decamer. The consensus sequence of the pentadecamer is 5′-TGCAnCTGTGnCCAG-3′ and it was characterized by an inner E-box motif 5′-CAnnTG-3′ except for the subgroups IGKV2 and IGKV4. An additional E-box was observed in subgroups IGKV3 and IGKV4 between the decamer and the TATA box (Figure 10).

## 4. Discussion

The *Macaca mulatta* (rhesus monkey) is one of the most widely used primate species in biomedical research and is used extensively as a model for studying human diseases, as it is evolutionarily close to humans [74,75,76]. In this study, an in silico research of the heavy and light chain IG genes was conducted based on the IMGT biocuration pipeline by using the “representative genome” (assembly Mmul_10) of the rhesus monkey from NCBI. Moreover, after a benchmarking of all the rhesus monkey assemblies available on NCBI, the Mmul_10 assembly [24] was found to be of better quality in terms of number of gaps and correct order of clusters for each IG locus. For example, within the assembly Mmul_8, the order of clusters from 5′ to 3′ for the IGH locus is: V-CLUSTER -> D-CLUSTER -> J-CLUSTER -> C-CLUSTER -> V-CLUSTER instead of V-CLUSTER -> D-CLUSTER -> J-CLUSTER -> C-CLUSTER. As another example for the IGH locus, the Mmul_8 assembly has 92 gaps, the Mmul_051212 assembly has 65 gaps, and the rheMacS_1.0 assembly has six gaps, while the Mmul_10 has only two gaps. It is worth noting that the current study focuses on the analysis of the Mmul_10 assembly, including the previously available rhesus monkey data, within IMGT^®^. However, a considerable IG genetic diversity in the form of allelic polymorphism and structural variation has been shown in previous genomic and germline gene inference studies [27,28], the results of which need to be taken into account for an improved overview of the IG gene repertoires of *Macaca mulatta*. Future work, within IMGT^®^, aims at analyzing the additional assemblies (for example RheMacS assembly [27] and the ASM545330 contigs [77]) based on the same model as well as incorporating the inferred alleles validated by the community. This will lead to the description of haplotypes taking into account the localization and the relative order of the genes in each locus in order to highlight new allelic polymorphisms and/or structural variations. This will also lead to more accurate gene assignments and calculations of somatic hypermutation (SHM) in this species. Extreme caution should be taken to correctly identify this genetic diversity.

Taking advantage of the IMGT biocuration pipeline, the IG germline repertoire and the IMGT^®^ reference directories were established according to IMGT^®^ nomenclature. The annotation of sequences, genes and structural data were integrated in the IMGT^®^ databases, tools and web resources. As a result of this effort, 597 IG genes and 908 IG alleles have been integrated into IMGT^®^. Despite the high similarity of the human and rhesus monkey genome, some differences have also been observed. Indeed, the genomic organization and characterization of IG genes highlighted that, based on the assembly Mmul_10 of the rhesus monkey and the data available on the IMGT/GENE-DB, the rhesus monkey has more V genes than human (excluding orphons). For example, the IMGT/GENE-DB contains 228 IGHV genes for rhesus monkeys versus 162 IGHV genes for humans; 149 IGLV genes for rhesus monkeys versus 79 IGLV genes for humans; 138 IGKV genes for rhesus monkeys versus 77 IGKV genes for humans. Consequently, this difference has an impact on the number of functional genes found in these species, 86 F IGHV genes for rhesus monkeys versus 57 F IGHV genes for humans; 73 F IGLV genes for rhesus monkeys versus 33 F IGLV genes for humans; 85 F IGKV genes for rhesus monkeys versus 41 F IGKV genes for humans. It would be interesting to study the genomic organization and characterization of IG genes in the other available *Macaca mulatta* assemblies, as well as in the genome of additional nonhuman primates to determine the proportion of genes for each species and track their evolution.

It was noted that, even though the germline CDR-IMGT lengths of the rhesus monkey vary between subgroups, and in some cases within a subgroup, certain CDR-IMGT lengths are much more frequent and are found in several subgroups. For example, the CDR-IMGT lengths [8.8.2] are frequently found in subgroups IGHV1, IGHV3, IGHV4, IGHV5, and IGHV7.

The heptamer and nonamer of the V, D and J genes recombination signals (RS) play a crucial role in the recombination process, their first three and last three nucleotides as well as the poly-A or poly-T tract of the nonamer are the specific characteristics used to identify them on genomic sequence (Figure 3). In order to determine whether a heptamer or nonamer is canonical and if it could be useful during the rearrangement process or not, the IMGT^®^ annotation rule for RS is as follows: if a heptamer or a nonamer is found in more than one functional gene in IMGT/GENE-DB for a given locus whatever the species, it is considered as canonical. If not, heptamers with at most one mutation and nonamers with at most two mutations compared with the corresponding consensus sequences in the locus are also considered as canonical. However, there are also exceptions where the heptamer and nonamer do not obey this rule and are found rearranged in cDNAs available in IMGT/LIGM-DB and generalist nucleotide databases (GenBank and ENA). For example, the J-HEPTAMER of the gene IGHJ2 (‘ggctgtg’ instead of ‘cactgtg’ or ‘caatgtg’) has been found rearranged in 159 cDNAs in IMGT/LIGM-DB [39] and the V-HEPTAMER of the allele IGLV8-125*01 (‘cacggcg’ instead of ‘cacagtg’) has also been found in cDNAs. Interestingly, the allele IGLV3-16*01, which is a pseudogene because there is no INIT-CODON (https://www.imgt.org/ligmdb/view?id=IMGT000062, accessed on 11 November 2021), is found rearranged in the cDNA accession number KCWN01002936.1 (https://www.ncbi.nlm.nih.gov/nuccore/KCWN01002936.1, accessed on 11 November 2021).

The identification of the core regulatory elements, located in the 5′UTR of the IG, reveals their conservation among eukaryotic species and would suggest their essential role in the transcription activity of the promoter. In this IG 5′UTR analysis of the rhesus monkey, all the core regulatory elements of the promoter region, that have been experimentally described in other eukaryotic species [29,30], have also been identified. Each IG chain, heavy and light, is characterized by different elements, however, a TATA box is always present (Figure 4, Figure 7 and Figure 10). This conserved A/T rich region upstream of the initiation codon could indicate and validate its important role in the transcriptional promoter activity. The rhesus monkey’s heavy chain promoters contain the octamer motif upstream the TATA box, whereas light chain promoters contain the identical sequence in an inverted and complementary form at the same position, with two additional conserved nucleotides in 5′ end (decamer). This element (octamer/decamer) is highly conserved in all of the subgroups in both heavy and light chain promoters, and its presence seems to be sufficient and necessary for the promoter activation [78,79]. Besides, experimental data in eukaryotes have shown that the octamer/decamer interacts and binds to central regulatory sequence-specific factors (Oct family proteins) in order to achieve a high transcriptional stimulation [78,79].

Heptanucleotide and pentadecamer are two core elements, characteristic for heavy chain and light chain promoters, respectively. It is observed that their sequences, in our dataset, are highly similar to the consensus sequences which were previously identified in experimental studies of eukaryotic species [80,81]. Although they are well aligned with these consensuses, in some cases, nucleotide alterations appear in their motifs, which provide uniqueness to the subgroup they are identified in. These two elements seem to remain conserved, to ensure a high rate of transcriptional activation, acting synergistically to the octamer or decamer elements [78,79]. However, the precise mechanism underlying this synergism remains to be elucidated for the *Macaca mulatta*.

Our IG promoter analysis revealed additional regulatory elements in the 5′ flanking sequences of immunoglobulin V genes, which could increase the rate of transcription [78]. These elements include a pyrimidine-rich region in heavy chain promoters (Figure 4), a CCCT element in light chain promoters (Figure 7 and Figure 10), and additional E-boxes and TATA boxes in both of them (heavy and light chains). More specifically, a CCCT element occurs in one or two repeats in the decamer flanking sequence of kappa and lambda chain UTRs. It has been shown experimentally in mouse cells that this element is very important for IG expression, and even in cases where the pentadecamer is absent it acts along with the decamer to stimulate transcription [79]. Moreover, a previous study [78] justifies that in the promoters where the CCCT element is missing, an E-box is detected and could fulfill the transcriptional activity, an observation that we also make in our analysis. For example, in the IGLV3 subgroup (Figure 7), the promoter region is lacking the pentadecamer, but the presence of two repeats of the CCCT element in the 5′ and 3′ flanking decamer and the E-box could probably activate the transcription.

Overall, our findings support the high degree of conservation of the fundamental regulatory promoter elements upstream the initiation codon of a V gene (ATG), as well as the existence of a distinct IG promoter organization. Despite this conservation, the consensus sequences and distances of the core elements of each subgroup vary significantly, indicating an auxiliary method for subgroup characterization and gene classification within subgroups. Notably, this observation along with the results of recent polymorphism studies in the human antibody upstream sequences could be valuable for the IG variable genes annotation process [29,30]. The elements identified and revealed in our study were previously described in the literature in other eukaryotic species and showed their necessary role in IG transcription, as well as the compensatory factor between elements, as their presence or absence could increase or decrease transcriptional activity, respectively [78,79]. Therefore, based on the Evo-devo scenarios and conservation, we could hypothesize that *Macaca mulatta* elements could have a similar fundamental role in IG regulation as in other eukaryotic species, however further in-depth experimental research needs to be done to validate this hypothesis. There is increasing evidence that 5′UTRs have a fundamental role in gene expression and further genomic studies could be performed to clarify and even explain these IG expression differences among individuals.

The study focuses on Mmul_10 assembly of rhesus monkey in order to provide to the scientific community rich and precise details about genes and alleles in this genome. For a given gene, up to 200 different information fields may be available for its annotation (IMGT labels of description, IMGT nomenclature, IMGT numbering, functionalities, isolate, reference sequence and sequence of the literature, description of alleles, protein display, colliers de perles, FR and CDR-length, etc.). This includes detailed characterisation of the regulatory elements of their promoters and their conventional recombination signals (RS), an overview of which is presented in this manuscript.

Taking into account the IG genetic diversity in the form of allelic and structural variations previously described [27,28], the approach described in this manuscript, and the resulting characterization of IG genes in Mmul_10, will be used as reference and model for the annotation of other published assemblies. The identification of position and order of genes in the IG loci of Mmul_10 provides a starting point for the characterization of allelic polymorphisms and of structural variations.

A comprehensive understanding of both innate and adaptive immune responses is essential for vaccine design and development [82,83]. Characterization of the *Macaca mulatta* IG loci in genomic assemblies, as shown in this study, provides important baseline information for this model species. However, the characterization of IG loci of additional assemblies and different animals will be required to generate a more complete IGH reference directory within IMGT, work that is currently underway.

## Figures and Tables

**Table 1:** Results obtained from the IG loci of *Macaca mulatta* (rhesus monkey).

Locus	IGH	IGL	IGK
Chromosome (orientation)	7 (REV)	10 (REV)	13 (FWD)
Size (kb)	1969	1301	1357
Number of V genes	208 (+20 unlocalized)	127 (+22 unlocalized)	110 (+28 unlocalized)
Number of D genes	45	0	0
Number of J genes	7	8	5
Number of C genes	8	8	1

Data available in IMGT Repertoire (IG and TR) http://www.imgt.org/IMGTrepertoire/, accessed on 11 November 2021 > Locus and genes > Locus descriptions > Locus description > IGH, ibid. IGL, ibid. IGK > Rhesus monkey.

**Table 2:** Number of V, D, J, and C genes per functionality, in the IGH, IGK and IGL loci of *Macaca mulatta*.

Genes	Functionality	Locus		
		IGH	IGL	IGK
V	F	79 (+7) *	72 (+1) *	76 (+9) *
	ORF	2 (+2) *	2	6 (+1) *
	P	140 (+5) *	74 (+1) *	47 (+8) *
D	F	44	/	/
	ORF	1	/	/
	P	0	/	/
J	F	7	6 (+1) *	5
	ORF	0	1 (+1) *	0
	P	0	0	0
C	F	8	6	1
	ORF	0	0	0
	P	0	2	0
Total		288	165	144

* An asterisk indicates that some genes have alleles with different functionalities: FUNCTIONAL or ORF (FO): IGHV3-186, IGHV3-103, IGKV2-86. FUNCTIONAL or PSEUDOGENE (FP): IGHV3-46, IGHV3-119, IGHV4-57, IGHV4-92, IGHV4-106, IGLV3-16, IGKV1-25, IGKV1-37, IGKV1-46, IGKV2-7, IGKV2-58, IGKV2-61, IGKV2-62, IGKV7-13. Data available in IMGT Repertoire (IG and TR) http://www.imgt.org/IMGTrepertoire/, accessed on 11 November 2021 > Locus and genes > Potential germline repertoires > IGH, ibid. IGL, ibid. IGK > Rhesus monkey.

**Table 3:** For each IGHV subgroup or clan, number of IGHV genes per functionality and, between parentheses, number of alleles.

IGHV Subgroup/Clan	Functionality	Total
IGHV1	11 F (13), 14 P (18)	25 (31)
IGHV2	5 F (6), 1 O (1), 1 P (1)	7 (8)
IGHV3	39 F (62), 1 O (2), 34 P (52), 2 FO (4), 2 FP (4)	78 (124)
IGHV4	19 F (25), 3 P (4), 3 FP (6)	25 (35)
IGHV5	2 F (4), 1 P (2)	3 (6)
IGHV6	1 F (1)	1 (1)
IGHV7	2 F (3), 12 P (14)	14 (17)
IGHV8	2 P (4)	2 (4)
IGHV(I)	6 P (9)	6 (9)
IGHV(II)	35 P (53)	35 (53)
IGHV(III)	32 P (48)	32 (48)
Total per functionality	79 F (114) + 2 O (3) + 140 P (205) + 2 FO (4) + 5 FP (10)	
Total number of genes (and alleles)		228 (336)

F: functional; O: ORF; P: pseudogene; FO: functional or ORF; FP: functional or pseudogene; the number of alleles is indicated in the parentheses. Data available in IMGT Repertoire (IG and TR) http://www.imgt.org/IMGTrepertoire/, accessed on 11 November 2021 > Locus and genes > Gene tables > IGHV > Nonhuman Primates > Rhesus monkey (*Macaca mulatta*).

**Table 4:** For each IGHD set, number of IGHD genes per functionality and, between parentheses, number of alleles.

IGHD Set	Functionality	Total
IGHD1	9 F (11)	9 (11)
IGHD2	7 F (8)	7 (8)
IGHD3	7 F (7)	7 (7)
IGHD4	7 F (7)	7 (7)
IGHD5	6 F (7), 1 O (1)	7 (8)
IGHD6	7 F (7)	7 (7)
IGHD7	1 F (1)	1 (1)
Total	44 F (48) + 1 O (1)	45 (49)

F: functional; O: ORF; the number of alleles is indicated in the parentheses. Data available in IMGT Repertoire (IG and TR) http://www.imgt.org/IMGTrepertoire/, accessed on 11 November 2021 > Locus and genes > Gene tables > IGHD > Nonhuman Primates > Rhesus monkey (*Macaca mulatta*).

**Table 5:** For each IGHJ set, number of IGHJ genes per functionality and, between parentheses, number of alleles.

IGHJ Set	Functionality
IGHJ1	1 F (2)
IGHJ2	1 F (1)
IGHJ3	1 F (1)
IGHJ4	1 F (1)
IGHJ5	2 F (4)
IGHJ6	1 F (1)
Total	7 F (10)

F: functional; the number of alleles is indicated in the parentheses. Data available in IMGT Repertoire (IG and TR) http://www.imgt.org/IMGTrepertoire/, accessed on 11 November 2021 > Locus and genes > Gene tables > IGHJ > Nonhuman Primates > Rhesus monkey (*Macaca mulatta*).

**Table 6:** For each IGHC isotype, number of IGHC genes per functionality and, between parentheses, number of alleles are shown.

IGHC Isotype	Functionality	Total
IGHM	1 FP (3)	1 (3)
IGHD	1 F (3)	1 (3)
IGHG	3 F (16), 1 FP (7)	4 (23)
IGHE	1 F (1)	1 (1)
IGHA	1 F (22)	1 (22)
Total	6 F (42) + 2 FP (10)	8 (52)

F: functional; FP: functional or pseudogene; the number of alleles is indicated in the parentheses. Data available in IMGT Repertoire (IG and TR) http://www.imgt.org/IMGTrepertoire/, accessed on 11 November 2021 > Locus and genes > Gene tables > IGHC > Nonhuman Primates > Rhesus monkey (*Macaca mulatta*).

**Table 7:** CDR-IMGT lengths distribution in each IGHV subgroup.

Subgroup	Germline [CDR1-IMGT.CDR2-IMGT.CDR3-IMGT] Length	Number of Genes *		
		F	ORF	In-Frame P
IGHV1	[8.8.2]	11	0	3
	[8.8.3]	0	0	1
IGHV2	[10.7.3]	5	1	0
IGHV3	[8.6.2]	2	0	0
	[8.7.2]	7	0	0
	[8.7.3]	0	1	0
	[8.8.0]	0	0	1
	[8.8.1]	0	0	1
	[8.8.2]	17	0	5
	[8.8.3]	2	0	3
	[8.9.2]	1	0	0
	[8.10.2]	12	1	2
IGHV4	[8.8.2]	8	0	0
	[9.8.2]	11	0	0
	[10.8.2]	2	0	0
IGHV5	[8.8.2]	2	0	1
IGHV6	[10.9.2]	1	0	0
IGHV7	[8.8.2]	2	0	7

* Only the first allele (*01) is taken into consideration. CDR-IMGT lengths are according to the IMGT unique numbering for V-REGION [70]. Data available in IMGT Repertoire (IG and TR) http://www.imgt.org/IMGTrepertoire/, accessed on 11 November 2021 > 2D and 3D structures > FR-IMGT and CDR-IMGT lengths (V-REGION and V-DOMAIN) > [CDR1-IMGT.CDR2-IMGT.] length per subgroup > IGHV > rhesus monkey (*Macaca mulatta*).

**Table 8:** For each IGLV subgroup, number of IGLV genes per functionality and, between parentheses, number of alleles is shown.

IGLV Subgroup/Clan	Functionality	Total
IGLV1	14 F (19), 5 P (12)	19 (31)
IGLV2	11 F (21), 5 P (6)	16 (27)
IGLV3	25 F (35), 1 O (3), 16 P (23), 1 FP (2)	43 (63)
IGLV4	3 F (5), 1 O (2), 1 P (2)	5 (9)
IGLV5	8 F (12), 8 P (15)	16 (27)
IGLV6	3 F (6), 2 P (2)	5 (8)
IGLV7	4 F (5), 5 P (5)	9 (10)
IGLV8	1 F (2)	1 (2)
IGLV9	1 F (1)	1 (1)
IGLV10	1 F (1), 1 P (1)	2 (2)
IGLV11	1 F (1)	1 (1)
IGLV(I)	18 P (24)	18 (24)
IGLV(II)	4 P (7)	4 (7)
IGLV(III)	3 P (4)	3 (4)
IGLV(IV)	5 P (8)	5 (8)
IGLV(V)	1 P (1)	1 (1)
Total per functionality	72 F (108) + 2 O (5) + 74 P (110) + 1 FP (2)	
Total number of genes (and alleles)		149 (225)

F: functional; O: ORF; P: pseudogene; FP: functional or pseudogene; the number of alleles is indicated in the parentheses. Data available in IMGT Repertoire (IG and TR) http://www.imgt.org/IMGTrepertoire/, accessed on 11 November 2021 > Locus and genes > Gene tables > IGLV > Nonhuman Primates > Rhesus monkey (*Macaca mulatta*).

**Table 9:** For each IGLJ set, number of IGLJ genes per functionality and, between parentheses, number of alleles are shown.

IGLJ Set	Functionality
IGLJ1	1 F (1)
IGLJ2	1 F (1)
IGLJ2A	1 F (1)
IGLJ3	1 F (1)
IGLJ4	1 O (1)
IGLJ5	1 F (1)
IGLJ6	1 F (1)
IGLJ7	1 FO (2)
Total per functionality	6 F (6) + 1 O (1) + 1 FO (2)
Total number of genes (and alleles)	8 (9)

F: functional; O: ORF; FO: functional or ORF; the number of alleles is indicated in the parentheses. Data available in IMGT Repertoire (IG and TR) http://www.imgt.org/IMGTrepertoire/, accessed on 11 November 2021 > Locus and genes > Gene tables > IGLJ > Nonhuman Primates > Rhesus monkey (*Macaca mulatta*).

**Table 10:** Number of IGLC genes per functionality and, between parentheses, number of alleles.

IGLC Set	Functionality
IGLC1	1 F (1)
IGLC2	1 F (2)
IGLC2A	1 F (1)
IGLC3	1 F (1)
IGLC4	1 P (2)
IGLC5	1 P (2)
IGLC6	1 F (2)
IGLC7	1 F (2)
Total per functionality	6 F (9) + 2 P (4)
Total number of genes (and alleles)	8 (13)

F: functional; P: pseudogene; the number of alleles is indicated in the parentheses. Data available in IMGT Repertoire (IG and TR) http://www.imgt.org/IMGTrepertoire/, accessed on 11 November 2021 > Locus and genes > Gene tables > IGLV > Nonhuman Primates > Rhesus monkey (*Macaca mulatta*).

**Table 11:** CDR-IMGT lengths distribution in each IGLV subgroup.

Subgroup	Germline [CDR1-IMGT.CDR2-IMGT.CDR3-IMGT] Length	Number of Genes *		
		F	ORF	In-Frame P
IGLV1	[9.3.9]	2	0	2
	[8.3.9]	12	0	0
IGLV2	[9.3.9]	11	0	1
IGLV3	[6.3.7]	5	0	0
	[6.3.8]	1	0	0
	[6.3.9]	18	1	1
	[6.3.12]	1	0	0
IGLV4	[7.7.7]	2	0	0
	[7.7.12]	1	1	0
IGLV5	[9.7.8]	7	0	1
	[9.7.9]	2	0	1
IGLV6	[8.3.8]	3	0	0
IGLV7	[9.3.8]	4	0	3
IGLV8	[9.3.8]	1	0	0
IGLV9	[7.8.12]	1	0	0
IGLV10	[8.3.9]	1	0	0
IGLV11	[9.7.8]	1	0	0

* Only the first allele (*01) is taken into consideration. CDR-IMGT lengths are according to the IMGT unique numbering for V-REGION [70]. Data available in IMGT Repertoire (IG and TR) http://www.imgt.org/IMGTrepertoire/, accessed on 11 November 2021 > 2D and 3D structures > FR-IMGT and CDR-IMGT lengths (V-REGION and V-DOMAIN) > [CDR1-IMGT.CDR2-IMGT.] length per subgroup > IGLV > rhesus monkey (*Macaca mulatta*).

**Table 12:** For each IGKV subgroup, number of IGKV genes per functionality and, between parentheses, number of alleles.

IGKV Subgroup/Clan	Functionality	Total
IGKV1	39 F (56), 1 O (1), 13 P (16), 3 FP (7)	56 (80)
IGKV2	21 F (28), 3 O (3), 21 P (29), 1 FO (2), 4 FP (10)	50 (72)
IGKV3	11 F (25), 1 O (1), 7 P (8)	19 (34)
IGKV4	1 F (2), 1 O (1), 2 P (4)	4 (7)
IGKV5	2 F (3)	2 (3)
IGKV6	2 F (2), 2P (2)	4 (4)
IGKV7	1 FP (2)	1 (2)
IGKV(II)	2 P (4)	2 (4)
Total per functionality	76 F (116) + 6 O (6) + 47 P (63) + 1 FO (2) + 8 FP (19)	
Total number of genes (and alleles)		138 (206)

F: functional; O: ORF; P: pseudogene; FO: functional or ORF; FP: functional or pseudogene; the number of alleles is indicated in the parentheses. Data available in IMGT Repertoire (IG and TR) http://www.imgt.org/IMGTrepertoire/, accessed on 11 November 2021 > Locus and genes > Gene tables > IGKV > Nonhuman Primates > Rhesus monkey (*Macaca mulatta*).

**Table 13:** For each IGKJ set, number of IGKJ genes per functionality and, between parentheses, number of alleles.

IGKJ Set	Functionality
IGKJ1	1 F (1)
IGKJ2	1 F (1)
IGKJ3	1 F (1)
IGKJ4	1 F (1)
IGKJ5	1 F (1)
Total per functionality	5 F (5)

F: functional; the number of alleles is indicated in the parentheses. Data available in IMGT Repertoire (IG and TR) http://www.imgt.org/IMGTrepertoire/, accessed on 11 November 2021 > Locus and genes > Gene tables > IGKJ > Nonhuman Primates > Rhesus monkey (*Macaca mulatta*).

**Table 14:** CDR-IMGT lengths distribution in each IGKV subgroup.

Subgroup	Germline [CDR1-IMGT.CDR2-IMGT.CDR3-IMGT] Lengths	Number of Genes *		
		F	ORF	In-Frame P
IGKV1	[6.3.7]	42	1	3
IGKV2	[11.3.7]	19	2	10
	[11.3.8]	0	0	2
	[12.3.7]	5	1	0
IGKV3	[6.3.4]	0	0	4
	[6.3.7]	11	1	0
	[7.3.7]	0	0	1
IGKV4	[12.3.7]	1	1	1
IGKV5	[6.3.7]	2	0	0
IGKV6	[6.3.7]	2	0	1
IGKV7	[10.3.7]	0	0	1

* Only the first allele (*01) is taken into consideration. CDR-IMGT lengths are according to the IMGT unique numbering for V-REGION [70]. Data available in IMGT Repertoire (IG and TR) http://www.imgt.org/IMGTrepertoire/, accessed on 11 November 2021 > 2D and 3D structures > FR-IMGT and CDR-IMGT lengths (V-REGION and V-DOMAIN) > [CDR1-IMGT.CDR2-IMGT.] length per subgroup > IGKV > rhesus monkey (*Macaca mulatta*).

## Data Availability

The IMGT^®^ software and data are provided to the academic users and NPO’s (Not for Profit Organization(s)) under the CC BY-NC-ND 4.0 license. Any other use of IMGT^®^ material, from the private sector, needs a financial arrangement with CNRS.

## Supplementary Materials

The following supporting information can be downloaded at: https://www.mdpi.com/article/10.3390/vaccines10030394/s1, Table S1: Information regarding the IGH RS consensus sequence by subgroup or set of the rhesus monkey (*Macaca mulatta*). Table S2: Information regarding the IGL RS consensus sequence by subgroup or set of the rhesus monkey (*Macaca mulatta*). Table S3: Information regarding the IGK RS consensus sequence by subgroup or set of the rhesus monkey (*Macaca mulatta*). Figure S1: Zoom of the J-C-CLUSTER of the rhesus monkey (*Macaca mulatta*) IGL locus.

## References

[1] Lefranc M.-P. (2014). Immunoglobulin and T Cell Receptor Genes: IMGT(^®^) and the Birth and Rise of Immunoinformatics. Front. Immunol..

[2] Lefranc M.-P., Lefranc G. (2001). The Immunoglobulin FactsBook.

[3] Lefranc M.-P., Lefranc G. (2001). The T Cell Receptor FactsBook.

[4] Tonegawa S. (1983). Somatic Generation of Antibody Diversity. Nature.

[5] Lefranc M.P. (2001). Nomenclature of the Human Immunoglobulin Heavy (IGH) Genes. Exp. Clin. Immunogenet..

[6] Lefranc M.P. (2001). Nomenclature of the Human Immunoglobulin Kappa (IGK) Genes. Exp. Clin. Immunogenet..

[7] Lefranc M.P. (2001). Nomenclature of the Human Immunoglobulin Lambda (IGL) Genes. Exp. Clin. Immunogenet..

[8] Lefranc M.-P., Lefranc G. (2020). Immunoglobulins or Antibodies: IMGT^®^Bridging Genes, Structures and Functions. Biomedicines.

[9] Tolbert W.D., Subedi G.P., Gohain N., Lewis G.K., Patel K.R., Barb A.W., Pazgier M. (2019). From Rhesus Macaque to Human: Structural Evolutionary Pathways for Immunoglobulin G Subclasses. mAbs.

[10] Verthelyi D., Wang V.W., Lifson J.D., Klinman D.M. (2004). CpG Oligodeoxynucleotides Improve the Response to Hepatitis B Immunization in Healthy and SIV-Infected Rhesus Macaques. AIDS.

[11] Gormus B.J., Blanchard J.L., Alvarez X.H., Didier P.J. (2004). Evidence for a Rhesus Monkey Model of Asymptomatic Tuberculosis. J. Med. Primatol..

[12] Shen Y., Shen L., Sehgal P., Zhou D., Simon M., Miller M., Enimi E.A., Henckler B., Chalifoux L., Sehgal N. (2001). Antiretroviral Agents Restore Mycobacterium-Specific T-Cell Immune Responses and Facilitate Controlling a Fatal Tuberculosis-like Disease in Macaques Coinfected with Simian Immunodeficiency Virus and Mycobacterium Bovis BCG. J. Virol..

[13] Shen Y., Zhou D., Chalifoux L., Shen L., Simon M., Zeng X., Lai X., Li Y., Sehgal P., Letvin N.L. (2002). Induction of an AIDS Virus-Related Tuberculosis-like Disease in Macaques: A Model of Simian Immunodeficiency Virus—Mycobacterium Coinfection. Infect. Immun..

[14] Ramesh A., Darko S., Hua A., Overman G., Ransier A., Francica J.R., Trama A., Tomaras G.D., Haynes B.F., Douek D.C. (2017). Structure and Diversity of the Rhesus Macaque Immunoglobulin Loci through Multiple De Novo Genome Assemblies. Front. Immunol..

[15] Sundling C., Li Y., Huynh N., Poulsen C., Wilson R., O’Dell S., Feng Y., Mascola J.R., Wyatt R.T., Karlsson Hedestam G.B. (2012). High-Resolution Definition of Vaccine-Elicited B Cell Responses against the HIV Primary Receptor Binding Site. Sci. Transl. Med..

[16] Roark R.S., Li H., Williams W.B., Chug H., Mason R.D., Gorman J., Wang S., Lee F.-H., Rando J., Bonsignori M. (2021). Recapitulation of HIV-1 Env-Antibody Coevolution in Macaques Leading to Neutralization Breadth. Science.

[17] Kong R., Duan H., Sheng Z., Xu K., Acharya P., Chen X., Cheng C., Dingens A.S., Gorman J., Sastry M. (2019). Antibody Lineages with Vaccine-Induced Antigen-Binding Hotspots Develop Broad HIV Neutralization. Cell.

[18] Martinez-Murillo P., Tran K., Guenaga J., Lindgren G., Àdori M., Feng Y., Phad G.E., Vázquez Bernat N., Bale S., Ingale J. (2017). Particulate Array of Well-Ordered HIV Clade C Env Trimers Elicits Neutralizing Antibodies That Display a Unique V2 Cap Approach. Immunity.

[19] Phad G.E., Pushparaj P., Tran K., Dubrovskaya V., Àdori M., Martinez-Murillo P., Vázquez Bernat N., Singh S., Dionne G., O’Dell S. (2020). Extensive Dissemination and Intraclonal Maturation of HIV Env Vaccine-Induced B Cell Responses. J. Exp. Med..

[20] Francica J.R., Sheng Z., Zhang Z., Nishimura Y., Shingai M., Ramesh A., Keele B.F., Schmidt S.D., Flynn B.J., Darko S. (2015). Analysis of Immunoglobulin Transcripts and Hypermutation Following SHIVAD8 Infection and Protein-plus-Adjuvant Immunization. Nat. Commun..

[21] Corbett K.S., Flynn B., Foulds K.E., Francica J.R., Boyoglu-Barnum S., Werner A.P., Flach B., O’Connell S., Bock K.W., Minai M. (2020). Evaluation of the MRNA-1273 Vaccine against SARS-CoV-2 in Nonhuman Primates. N. Engl. J. Med..

[22] Deng W., Bao L., Gao H., Xiang Z., Qu Y., Song Z., Gong S., Liu J., Liu J., Yu P. (2020). Ocular Conjunctival Inoculation of SARS-CoV-2 Can Cause Mild COVID-19 in Rhesus Macaques. Nat. Commun..

[23] Lefranc M.-P., Giudicelli V., Duroux P., Jabado-Michaloud J., Folch G., Aouinti S., Carillon E., Duvergey H., Houles A., Paysan-Lafosse T. (2015). IMGT^®^, the International ImMunoGeneTics Information System^®^ 25 Years On. Nucleic Acids Res..

[24] Warren W.C., Harris R.A., Haukness M., Fiddes I.T., Murali S.C., Fernandes J., Dishuck P.C., Storer J.M., Raveendran M., Hillier L.W. (2020). Sequence Diversity Analyses of an Improved Rhesus Macaque Genome Enhance Its Biomedical Utility. Science.

[25] Gibbs R.A., Rogers J., Katze M.G., Bumgarner R., Weinstock G.M., Mardis E.R., Remington K.A., Strausberg R.L., Venter J.C., Rhesus Macaque Genome Sequencing and Analysis Consortium (2007). Evolutionary and Biomedical Insights from the Rhesus Macaque Genome. Science.

[26] Watson C.T., Steinberg K.M., Huddleston J., Warren R.L., Malig M., Schein J., Willsey A.J., Joy J.B., Scott J.K., Graves T.A. (2013). Complete Haplotype Sequence of the Human Immunoglobulin Heavy-Chain Variable, Diversity, and Joining Genes and Characterization of Allelic and Copy-Number Variation. Am. J. Hum. Genet..

[27] He Y., Luo X., Zhou B., Hu T., Meng X., Audano P.A., Kronenberg Z.N., Eichler E.E., Jin J., Guo Y. (2019). Long-Read Assembly of the Chinese Rhesus Macaque Genome and Identification of Ape-Specific Structural Variants. Nat. Commun..

[28] Vázquez Bernat N., Corcoran M., Nowak I., Kaduk M., Castro Dopico X., Narang S., Maisonasse P., Dereuddre-Bosquet N., Murrell B., Karlsson Hedestam G.B. (2021). Rhesus and Cynomolgus Macaque Immunoglobulin Heavy-Chain Genotyping Yields Comprehensive Databases of Germline VDJ Alleles. Immunity.

[29] Mikocziova I., Gidoni M., Lindeman I., Peres A., Snir O., Yaari G., Sollid L.M. (2020). Polymorphisms in Immunoglobulin Heavy Chain Variable Genes and Their Upstream Regions. bioRxiv.

[30] Zhu Y., Yang X., Ma C., Tang H., Wang Q., Guan J., Xie W., Chen S., Chen Y., Wang M. (2021). Antibody Upstream Sequence Diversity and Its Biological Implications Revealed by Repertoire Sequencing. J. Genet. Genom..

[31] Steri M., Idda M.L., Whalen M.B., Orrù V. (2018). Genetic Variants in MRNA Untranslated Regions. WIREs RNA.

[32] Lane J., Duroux P., Lefranc M.-P. (2010). From IMGT-ONTOLOGY to IMGT/LIGMotif: The IMGT Standardized Approach for Immunoglobulin and T Cell Receptor Gene Identification and Description in Large Genomic Sequences. BMC Bioinform..

[33] Folch G., Jabado-Michaloud J., Bellahcene F., Regnier L., Giudicelli V., Lefranc M.-P. (2009). IMGT/Automat: The Strategy for the Annotation of Human and Mouse CDNA Nucleotide Sequences of IG and TR. Nat. Preced..

[34] Giudicelli V., Lefranc M.-P. (2012). IMGT-ONTOLOGY 2012. Front. Genet..

[35] Pégorier P., Bertignac M., Chentli I., Nguefack Ngoune V., Folch G., Jabado-Michaloud J., Hadi-Saljoqi S., Giudicelli V., Duroux P., Lefranc M.-P. (2020). IMGT^®^ Biocuration and Comparative Study of the T Cell Receptor Beta Locus of Veterinary Species Based on *Homo sapiens* TRB. Front. Immunol..

[36] Kitts P.A., Church D.M., Thibaud-Nissen F., Choi J., Hem V., Sapojnikov V., Smith R.G., Tatusova T., Xiang C., Zherikov A. (2016). Assembly: A Resource for Assembled Genomes at NCBI. Nucleic Acids Res..

[37] Brochet X., Lefranc M.-P., Giudicelli V. (2008). IMGT/V-QUEST: The Highly Customized and Integrated System for IG and TR Standardized V-J and V-D-J Sequence Analysis. Nucleic Acids Res..

[38] Lemoine F., Correia D., Lefort V., Doppelt-Azeroual O., Mareuil F., Cohen-Boulakia S., Gascuel O. (2019). NGPhylogeny.Fr: New Generation Phylogenetic Services for Non-Specialists. Nucleic Acids Res..

[39] Giudicelli V., Duroux P., Ginestoux C., Folch G., Jabado-Michaloud J., Chaume D., Lefranc M.-P. (2006). IMGT/LIGM-DB, the IMGT Comprehensive Database of Immunoglobulin and T Cell Receptor Nucleotide Sequences. Nucleic Acids Res..

[40] Giudicelli V., Chaume D., Lefranc M.-P. (2005). IMGT/GENE-DB: A Comprehensive Database for Human and Mouse Immunoglobulin and T Cell Receptor Genes. Nucleic Acids Res..

[41] Kaas Q., Ruiz M., Lefranc M.-P. (2004). IMGT/3Dstructure-DB and IMGT/StructuralQuery, a Database and a Tool for Immunoglobulin, T Cell Receptor and MHC Structural Data. Nucleic Acids Res..

[42] Alamyar E., Duroux P., Lefranc M.-P., Giudicelli V. (2012). IMGT(^®^) Tools for the Nucleotide Analysis of Immunoglobulin (IG) and T Cell Receptor (TR) V-(D)-J Repertoires, Polymorphisms, and IG Mutations: IMGT/V-QUEST and IMGT/HighV-QUEST for NGS. Methods Mol. Biol..

[43] Ehrenmann F., Kaas Q., Lefranc M.-P. (2010). IMGT/3Dstructure-DB and IMGT/DomainGapAlign: A Database and a Tool for Immunoglobulins or Antibodies, T Cell Receptors, MHC, IgSF and MhcSF. Nucleic Acids Res..

[44] Lefranc M.-P. (2011). IMGT Collier de Perles for the Variable (V), Constant (C), and Groove (G) Domains of IG, TR, MH, IgSF, and MhSF. Cold Spring Harb. Protoc..

[45] Ehrenmann F., Giudicelli V., Duroux P., Lefranc M.-P. (2011). IMGT/Collier de Perles: IMGT Standardized Representation of Domains (IG, TR, and IgSF Variable and Constant Domains, MH and MhSF Groove Domains). Cold Spring Harb. Protoc..

[46] Bemark M., Liberg D., Leanderson T. (1998). Conserved Sequence Elements in K Promoters from Mice and Humans: Implications for Transcriptional Regulation and Repertoire Expression. Immunogenetics.

[47] Falkner F.G., Zachau H.G. (1984). Correct Transcription of an Immunoglobulin Kappa Gene Requires an Upstream Fragment Containing Conserved Sequence Elements. Nature.

[48] Haino M., Hayashida H., Miyata T., Shin E.K., Matsuda F., Nagaoka H., Matsumura R., Taka-ishi S., Fukita Y., Fujikura J. (1994). Comparison and Evolution of Human Immunoglobulin VH Segments Located in the 3′ 0.8-Megabase Region. Evidence for Unidirectional Transfer of Segmental Gene Sequences. J. Biol. Chem..

[49] Parslow T.G., Blair D.L., Murphy W.J., Granner D.K. (1984). Structure of the 5′ Ends of Immunoglobulin Genes: A Novel Conserved Sequence. Proc. Natl. Acad. Sci. USA.

[50] Eaton S., Calame K. (1987). Multiple DNA Sequence Elements Are Necessary for the Function of an Immunoglobulin Heavy Chain Promoter. Proc. Natl. Acad. Sci. USA.

[51] Grosschedl R., Baltimore D. (1985). Cell-Type Specificity of Immunoglobulin Gene Expression Is Regulated by at Least Three DNA Sequence Elements. Cell.

[52] Lis M., Walther D. (2016). The Orientation of Transcription Factor Binding Site Motifs in Gene Promoter Regions: Does It Matter?. BMC Genom..

[53] Yella V.R., Kumar A., Bansal M. (2018). Identification of Putative Promoters in 48 Eukaryotic Genomes on the Basis of DNA Free Energy. Sci. Rep..

[54] Henson R., Cetto L. (2005). The MATLAB Bioinformatics Toolbox. Encycl. Genet. Genom. Proteom. Bioinform..

[55] Vlachakis D., Papageorgiou L., Papadaki A., Georga M., Kossida S., Eliopoulos E. (2020). An Updated Evolutionary Study of the Notch Family Reveals a New Ancient Origin and Novel Invariable Motifs as Potential Pharmacological Targets. PeerJ.

[56] Papageorgiou L., Loukatou S., Sofia K., Maroulis D., Vlachakis D. (2016). An Updated Evolutionary Study of Flaviviridae NS3 Helicase and NS5 RNA-Dependent RNA Polymerase Reveals Novel Invariable Motifs as Potential Pharmacological Targets. Mol. Biosyst..

[57] Madeira F., Park Y.M., Lee J., Buso N., Gur T., Madhusoodanan N., Basutkar P., Tivey A.R.N., Potter S.C., Finn R.D. (2019). The EMBL-EBI Search and Sequence Analysis Tools APIs in 2019. Nucleic Acids Res..

[58] Katoh K., Rozewicki J., Yamada K.D. (2019). MAFFT Online Service: Multiple Sequence Alignment, Interactive Sequence Choice and Visualization. Brief. Bioinform..

[59] Dereeper A., Guignon V., Blanc G., Audic S., Buffet S., Chevenet F., Dufayard J.-F., Guindon S., Lefort V., Lescot M. (2008). Phylogeny.Fr: Robust Phylogenetic Analysis for the Non-Specialist. Nucleic Acids Res..

[60] Waterhouse A.M., Procter J.B., Martin D.M.A., Clamp M., Barton G.J. (2009). Jalview Version 2-a Multiple Sequence Alignment Editor and Analysis Workbench. Bioinformatics.

[61] Farré D., Roset R., Huerta M., Adsuara J.E., Roselló L., Albà M.M., Messeguer X. (2003). Identification of Patterns in Biological Sequences at the ALGGEN Server: PROMO and MALGEN. Nucleic Acids Res..

[62] Latchman D. (2007). Gene Regulation.

[63] Matys V., Fricke E., Geffers R., Gössling E., Haubrock M., Hehl R., Hornischer K., Karas D., Kel A.E., Kel-Margoulis O.V. (2003). TRANSFAC: Transcriptional Regulation, from Patterns to Profiles. Nucleic Acids Res..

[64] Stothard P. (2000). The Sequence Manipulation Suite: JavaScript Programs for Analyzing and Formatting Protein and DNA Sequences. Biotechniques.

[65] Thomas-Chollier M., Hufton A., Heinig M., O’Keeffe S., Masri N.E., Roider H.G., Manke T., Vingron M. (2011). Transcription Factor Binding Predictions Using TRAP for the Analysis of ChIP-Seq Data and Regulatory SNPs. Nat. Protoc..

[66] Lee T.-Y., Chang W.-C., Hsu J.B.-K., Chang T.-H., Shien D.-M. (2012). GPMiner: An Integrated System for Mining Combinatorial Cis-Regulatory Elements in Mammalian Gene Group. BMC Genom..

[67] Solovyev V.V., Shahmuradov I.A., Salamov A.A. (2010). Identification of Promoter Regions and Regulatory Sites. Methods Mol. Biol..

[68] Bible J.M., Howard W., Robbins H., Dunn-Walters D.K. (2003). IGHV1, IGHV5 and IGHV7 Subgroup Genes in the Rhesus Macaque. Immunogenetics.

[69] Helmuth E.F., Letvin N.L., Margolin D.H. (2000). Germline Repertoire of the Immunoglobulin V(H)3 Family in Rhesus Monkeys. Immunogenetics.

[70] Lefranc M.-P., Pommié C., Ruiz M., Giudicelli V., Foulquier E., Truong L., Thouvenin-Contet V., Lefranc G. (2003). IMGT Unique Numbering for Immunoglobulin and T Cell Receptor Variable Domains and Ig Superfamily V-like Domains. Dev. Comp. Immunol..

[71] Crooks G.E., Hon G., Chandonia J.-M., Brenner S.E. (2004). WebLogo: A Sequence Logo Generator. Genome Res..

[72] Howard W.A., Bible J.M., Finlay-Dijsselbloem E., Openshaw S., Dunn-Walters D.K. (2005). Immunoglobulin Light-Chain Genes in the Rhesus Macaque II: Lambda Light-Chain Germline Sequences for Subgroups IGLV1, IGLV2, IGLV3, IGLV4 and IGLV5. Immunogenetics.

[73] Howard W.A., Bible J.M., Finlay-Dijsselbloem E., Openshaw S., Dunn-Walters D.K. (2005). Immunoglobulin Light-Chain Genes in the Rhesus Macaque I: Kappa Light-Chain Germline Sequences for Subgroups IGKV1, IGKV and IGKV3. Immunogenetics.

[74] Messaoudi I., Estep R., Robinson B., Wong S.W. (2011). Nonhuman Primate Models of Human Immunology. Antioxid. Redox Signal..

[75] Phad G.E., Bernat N.V., Feng Y., Ingale J., Murillo P.A.M., O’Dell S., Li Y., Mascola J.R., Sundling C., Wyatt R.T. (2015). Diverse Antibody Genetic and Recognition Properties Revealed Following HIV-1 Envelope Glycoprotein Immunization. The J. Immunol..

[76] Cottrell C.A., van Schooten J., Bowman C.A., Yuan M., Oyen D., Shin M., Morpurgo R., van der Woude P., van Breemen M., Torres J.L. (2020). Mapping the Immunogenic Landscape of Near-Native HIV-1 Envelope Trimers in Non-Human Primates. PLoS Pathog..

[77] Cirelli K.M., Carnathan D.G., Nogal B., Martin J.T., Rodriguez O.L., Upadhyay A.A., Enemuo C.A., Gebru E.H., Choe Y., Viviano F. (2019). Slow Delivery Immunization Enhances HIV Neutralizing Antibody and Germinal Center Responses via Modulation of Immunodominance. Cell.

[78] Sigvardsson M., Bemark M., Leanderson T. (1995). Pentadecamer-Binding Proteins: Definition of Two Independent Protein-Binding Sites Needed for Functional Activity. Mol. Cell Biol..

[79] Högbom E., Magnusson A.C., Leanderson T. (1991). Functional Modularity in the SP6 Kappa Promoter. Nucleic Acids Res..

[80] Landolfi N.F., Yin X.M., Capra J.D., Tucker P.W. (1988). A Conserved Heptamer Upstream of the IgH Promoter Region Octamer Can Be the Site of a Coordinate Protein-DNA Interaction. Nucleic Acids Res..

[81] Falkner F.G., Neumann E., Zachau H.G. (1984). Tissue Specificity of the Initiation of Immunoglobulin Kappa Gene Transcription. Hoppe Seylers Z. Physiol. Chem..

[82] Galinski M.R., Lapp S.A., Peterson M.S., Ay F., Joyner C.J., LE Roch K.G., Fonseca L.L., Voit E.O., MAHPIC CONSORTIUM (2018). *Plasmodium knowlesi*: A Superb in Vivo Nonhuman Primate Model of Antigenic Variation in Malaria. Parasitology.

[83] Itell H.L., Nelson C.S., Martinez D.R., Permar S.R. (2017). Maternal Immune Correlates of Protection against Placental Transmission of Cytomegalovirus. Placenta.

