# Supplementary material for: IMGT® Biocuration and Analysis of the Rhesus Monkey IG Loci
Source: Vaccines (Basel). 2022 Mar 3;10(3):394. doi: 10.3390/vaccines10030394 (PMC8950363; doi:10.3390/vaccines10030394)
Supplement: Supplementary file 1 [file vaccines-10-00394-s001.zip › vaccines-1483231-supplementary.pdf]

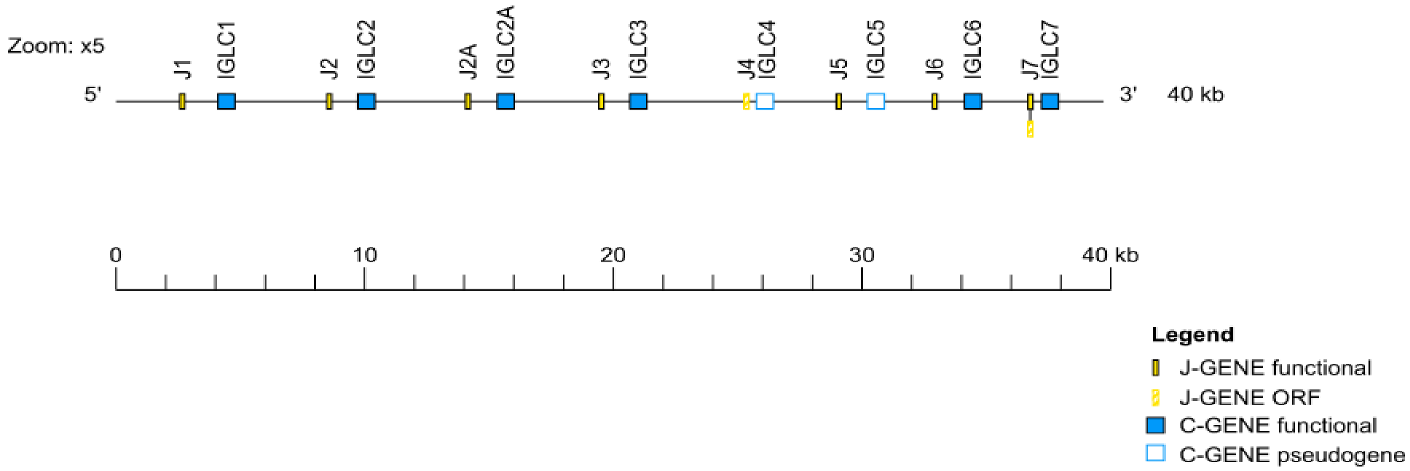

Figure S1. Zoom x5 of the IGL J-C-CLUSTER which comprises 8 cassettes indicated by the numbers 1 to 7 (IGLJ1-IGLC1, IGLJ2-IGLC2, IGLJ2A-IGLC2A, IGLJ3-IGLC3, IGLJ4-IGLC4, IGLJ5-IGLC5, IGLJ6-IGLC6 and IGLJ7-IGLC7 respectively). Data available in IMGT Repertoire (IG and TR) http://www.imgt.org/IMGTrepertoire/ (accessed on the 1st March 2022)> Locus and genes > Locus representations > IGL > Rhesus monkey.

Table S1. a. IGHV RS V-HEPTAMER and V-NONAMER consensus sequences for all functional IGHV and per IGHV subgroup of the *Macaca mulatta* (rhesus monkey).

**IGHV subgroups**	**V-HEPTAMER**							**V-NONAMER**								
**All functional IGHV**	**c**	**a**	**c**	**a**	**g**	**t**	**g**	**a**	**c**	**a**	**c**	**a**	**a**	a	c	c
IGHV1	-	-	-	-	-	-	-	t	-	-	g	-	-	-	-	-
IGHV2	-	-	-	-	-	a	-	-	-	-	a	g	-	-	-	-
IGHV3	-	-	-	-	-	-	-	-	-	-	-	-	-	-	-	-
IGHV4	-	-	-	-	-	-	-	-	-	-	-	-	-	-	-	-
IGHV5	-	-	-	-	-	-	-	c	-	-	a	-	-	-	-	-
IGHV6	-	-	-	-	-	-	-	-	-	-	-	-	-	-	-	-
IGHV7	-	-	-	-	-	-	-									

An "-" is marked when the nucleotide is the same as the IGHV consensus. Data extracted from http://www.imgt.org/genedb/. (accessed on the 1st March 2022)

Table S1. b. IGHJ RS J-HEPTAMER and J-NONAMER consensus sequences for all functional IGHJ and per IGHJ set of the *Macaca mulatta* (rhesus monkey).

**IGHJ sets**	**J-HEPTAMER**							**J-NONAMER**								
**All functional IGHJ**	**c**	**a**	**a**	**t**	**g**	**t**	**g**	**g**	**g**	**t**	**t**	**t**	**t**	t	g	t
IGHJ1	-	-	c	-	-	-	-	-	-	-	-	-	c	-	-	c
IGHJ2	g	g	c	-	-	-	-	t	-	-	-	-	-	-	-	-
IGHJ3	-	c	c	-	-	-	-	-	-	-	-	-	g	-	-	-
IGHJ4	-	-	-	-	-	-	-	-	-	-	-	-	-	-	-	-
IGHJ5	-	-	-	-	-	-	-	a	-	-	-	c	-	-	-	c
IGHJ6	-	-	t	-	-	-	-	-	-	-	-	-	-	-	-	-

An "-" is marked when the nucleotide is the same as the consensus. Data extracted from http://www.imgt.org/genedb/. (accessed on the 1st March 2022)

Table S1. c. IGHD RS 5′D-HEPTAMER and 5′D-NONAMER consensus sequences for all functional IGHD and per IGHD set of the *Macaca mulatta* (rhesus monkey).

IGHD sets	5′D-HEPTAMER							5′D-NONAMER								
**All functional IGHD**	**c**	**a**	**c**	**t**	**g**	**t**	**g**	**g**	**g**	**t**	**t**	**t**	**t**	t	g	t
IGHD1	-	-	-	g	-	-	-	-	-	a	-	-	c	-	-	a
IGHD2	-	-	-	-	-	-	-	-	-	a	-	-	-	-	-	-
IGHD3	-	-	-	-	-	-	-	-	-	-	-	-	g	g	a	g
IGHD4	t	-	-	-	-	-	-	-	c	-	-	-	-	-	-	-
IGHD5	g	g	-	-	-	-	-	-	-	-	-	a	-	-	-	-
IGHD6	-	-	-	a	-	-	-	-	-	-	-	-	c	-	-	a
IGHD7	-	-	-	-	-	-	-	-	-	-	-	-	-	g	-	c

An "-" is marked when the nucleotide is the same as the consensus. Data extracted from http://www.imgt.org/genedb/. (accessed on the 1st March 2022)

Table S1. d. IGHD RS 3′D-HEPTAMER and 3′D-NONAMER consensus sequences for all functional IGHD and per IGHD set of the *Macaca mulatta* (rhesus monkey).

IGHD sets	3′D-HEPTAMER							3′D-NONAMER								
**All functional IGHD**	**c**	**a**	**c**	**a**	**g**	**t**	**g**	**t**	**c**	**a**	**a**	**a**	**a**	a	c	c
IGHD1	-	-	-	t	-	-	-	-	-	c	-	-	-	-	-	-
IGHD2	-	-	-	-	-	-	-	-	-	c	c	-	-	-	g	-
IGHD3	-	-	-	-	-	-	-	-	-	-	-	-	-	-	-	-
IGHD4	-	-	-	-	-	-	-									
IGHD5	-	-	-	-	-	-	-	g	-	-	g	c	-	-	-	-
IGHD6	-	-	-	-	-	-	-	c	-	-	g	-	-	-	-	-
IGHD7	-	-	-	-	-	-	-	g	-	-	-	-	-	-	-	-

An "-" is marked when the nucleotide is the same as the consensus. Data extracted from http://www.imgt.org/genedb/. (accessed on the 1st March 2022)

Table S2. a. IGLV RS V-HEPTAMER and V-NONAMER consensus sequences for All functional IGLV and per IGLV subgroup of the *Macaca mulatta* (rhesus monkey) rhesus monkey.

IGLV subgroups	V-HEPTAMER							V-NONAMER								
**All functional IGLV**	**c**	**a**	**c**	**a**	**g**	**t**	**g**	**a**	**c**	**a**	**a**	**a**	**a**	a	c	c
IGLV1	-	-	-	-	-	-	-	-	-	-	-	g	-	-	-	-
IGLV2	-	-	-	-	-	-	-	-	-	c	-	-	-	-	-	-
IGLV3	-	-	-	-	-	-	-	-	-	-	g	-	-	-	-	-
IGLV4	-	-	-	-	-	-	-	-	-	-	-	-	-	t	-	-
IGLV5	-	-	-	-	-	-	-	-	-	-	-	-	-	-	-	-
IGLV6	-	-	-	-	-	-	a	-	-	-	-	g	-	-	-	t
IGLV7	-	-	-	-	-	-	-	-	-	-	t	-	-	-	-	-
IGLV8								-	-	c	-	-	-	-	-	-
IGLV9	-	-	-	-	-	-	-	-	-	-	-	-	-	-	-	-
IGLV10	-	-	-	-	-	-	-	-	t	-	-	-	-	-	-	t
IGLV11	-	-	-	-	-	-	-	-	-	-	-	-	-	-	-	-

An "-" is marked when the nucleotide is the same as the consensus. Data extracted from http://www.imgt.org/genedb/. (accessed on the 1st March 2022)

Table S2. b. IGLJ RS J-HEPTAMER and J-NONAMER consensus sequences for All functional IGLJ and per IGLJ set of the *Macaca mulatta* (rhesus monkey).

IGLJ sets	J-HEPTAMER							J-NONAMER								
**All functional IGLJ**	**c**	**a**	**c**	**a**	**g**	**t**	**g**	**g**	**g**	**t**	**t**	**t**	**t**	t	g	t
IGLJ1	-	-	-	t	-	-	-	-	-	-	-	-	-	g	-	-
IGLJ2	-	-	-	-	-	-	-	-	-	-	-	-	-	-	-	-
IGLJ2A	-	-	-	-	-	-	-	-	-	-	-	-	-	-	-	-
IGLJ3	-	-	-	-	-	-	-	c	-	-	-	-	-	-	-	-
IGLJ5	-	-	-	-	-	c	a	-	-	-	-	-	-	-	-	-
IGLJ6	-	-	-	-	-	-	-	-	-	-	-	-	g	-	-	-
IGLJ7	-	-	-	t	-	-	-	-	-	-	-	-	g	-	-	-

An "-" is marked when the nucleotide is the same as the consensus. Data extracted from http://www.imgt.org/genedb/. (accessed on the 1st March 2022)

Table S3. a. IGKV RS V-HEPTAMER and V-NONAMER consensus sequences for all functional IGKV and per IGKV subgroup of the *Macaca mulatta* (rhesus monkey).

IGKV subgroups	V-HEPTAMER							V-NONAMER								
**All functional IGKV**	**c**	**a**	**c**	**a**	**g**	**t**	**g**	**a**	**c**	**a**	**a**	**a**	**a**	a	c	c
IGKV1	-	-	-	-	-	-	-	-	-	-	t	-	-	-	-	-
IGKV2	-	-	-	-	-	-	-	-	-	-	g	-	-	-	-	-
IGKV3	-	-	-	-	-	-	-	-	-	-	-	-	-	-	-	-
IGKV4	-	-	-	-	-	-	-	-	-	-	c	-	-	-	-	-
IGKV5	-	-	-	-	-	-	-	-	-	-	-	-	-	-	-	-
IGKV6	-	-	-	-	c	-	-	-	-	-	-	-	-	-	-	t
IGKV7	-	-	-	-	-	-	-	-	-	-	-	-	-	-	-	-

An "-" is marked when the nucleotide is the same as the consensus. Data extracted from http://www.imgt.org/genedb/. (accessed on the 1st March 2022)

Table S3. b. IGKJ RS J-HEPTAMER and J-NONAMER consensus sequences for all functional IGKJ and per IGKJ set of the *Macaca mulatta* (rhesus monkey).

IGKJ sets	J-HEPTAMER							J-NONAMER								
**All functional IGKJ**	**c**	**a**	**c**	**t**	**g**	**t**	**g**	**g**	**g**	**t**	**t**	**t**	**t**	t	g	t
IGKJ1	-	-	-	-	-	-	-	-	-	-	-	-	-	-	-	-
IGKJ2	-	-	t	-	-	-	-	a	-	-	-	-	-	-	-	-
IGKJ3	-	-	-	-	-	-	-	-	-	-	-	-	-	-	-	-
IGKJ4	-	-	-	-	-	-	-	-	-	-	-	-	-	-	-	-
IGKJ5	t	-	-	-	-	-	-	-	a	-	-	-	-	-	-	-

An "-" is marked when the nucleotide is the same as the consensus. Data extracted from http://www.imgt.org/genedb/. (accessed on the 1st March 2022)
